# Development of an Optoelectronic Sensor for Detecting and Classifying Fruit Fly (Diptera: Tephritidae) for Use in Real-Time Intelligent Traps

**DOI:** 10.3390/s19051254

**Published:** 2019-03-12

**Authors:** Fabiano Sandrini Moraes, Dori Edson Nava, Tiago Scheunemann, Vagner Santos da Rosa

**Affiliations:** 1 Computational Sciences Center, Federal University of Rio Grande—FURG, Rio Grande, RS 96230-000, Brazil; vagner.rosa@furg.br; 2 South Rio Grande do Sul Federal Institute—IFSUL, Pelotas, RS 96015-360, Brazil; 3 Embrapa Temperate Agriculture, Entomology Laboratory, Pelotas, RS 96010-971, Brazil; dori.edson-nava@embrapa.br; 4 Phytosanitary Graduate Program of the Federal University of Pelotas—UFPel, Pelotas, RS 96010-900, Brazil; tiagoscheunemann@gmail.com

**Keywords:** fruit fly, optoelectronic sensors, classification of automated insects, signal processing, precision agriculture, pest monitoring

## Abstract

Fruit flies (Diptera: Tephritidae) cause losses to world fruit growing. For a fast and effective control of the pest, it is necessary to identify the species and their populations. Thus, we developed an infrared optoelectronic sensor using phototransistors to capture the signal of the partial occlusion of the infrared light caused by the beating of the fly wings. Laboratory experiments were conducted using the sensor to capture the wing beat signal of *A. fraterculus* and *C. capitata*. The captured signals were used to obtain the characteristics of the flies’ wing beats frequency and for a production of a dataset made available as one of the results of this work. For the passage detection, we developed the algorithm of detection of events of passage (PEDA) that uses the root mean square (RMS) value of a sliding window applied to the signal compared to a threshold value. We developed the algorithm of detection of events of passage (CAEC) that uses the techniques of autocorrelation and Fourier transform for the extraction of the characteristics of the wings’ beat signal. The results demonstrate that it is possible to use the sensor for the development of an intelligent trap with detection and classification in real time for *A. fraterculus* and *C. capitata* using the wing beat frequency obtained by the developed sensor.

## 1. Introduction

Fruit flies (Diptera: Tephritidae) are considered to be the main pests affecting fruit growing worldwide causing direct and indirect damages in production. In Europe alone, these losses and impacts are estimated to cost at least EUR 12 billion per year [1]. In Brazil, data from the Ministry of Agriculture, Livestock and Supply estimates that, in 2015, the damage caused by fruit flies to Brazilian farmers with production losses and costs in pest control was US$ 120 million [2]. The presence of flies also makes it impossible to export fresh fruit to more demanding and profitable markets such as Japan, the United States and Chile [2]. The main species of fruit flies of economic importance in Brazil belong to three genera: *Anastrepha*, *Bactrocera* and *Ceratitis*. Among the species of fruit flies present in Brazil, those that present quarantine restrictions for importing countries are: *Anastrepha fraterculus* (Wiedemann, 1830), *Anastrepha obliqua* (Macquart, 1835), *Anastrepha grandis* (Macquart, 1846), *Ceratitis capitata* (Wiedemann, 1824) and *Bactrocera carambolae* (Drew and Hancock, 1994) [3].

**Figure 1:**
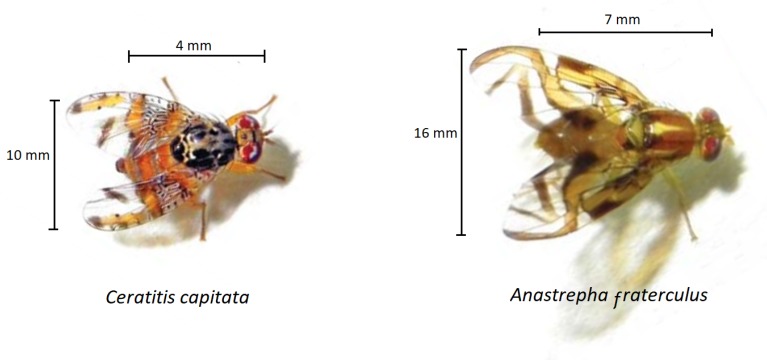
Fruit flies *C. capita* and *A. fraterculus*.

Fruit flies have wide geographical distribution and a large number of hosts. In the southern region of Brazil, *C. capitata* and *A. fraterculus* (Figure 1) develop in several fruit trees and can not make production feasible if control measures are not adopted [4].

Fruit growers control the fruit flies in an unruly manner using insecticides in the form of bait-toxic or by cover without knowledge of the infecting species, levels of infestation and distribution of hosts. This type of control has several undesirable consequences such as environmental impact, reduction in fruit quality, export restrictions due to the presence of chemical residues and an increase in the cost of production [4].

**Figure 2:**
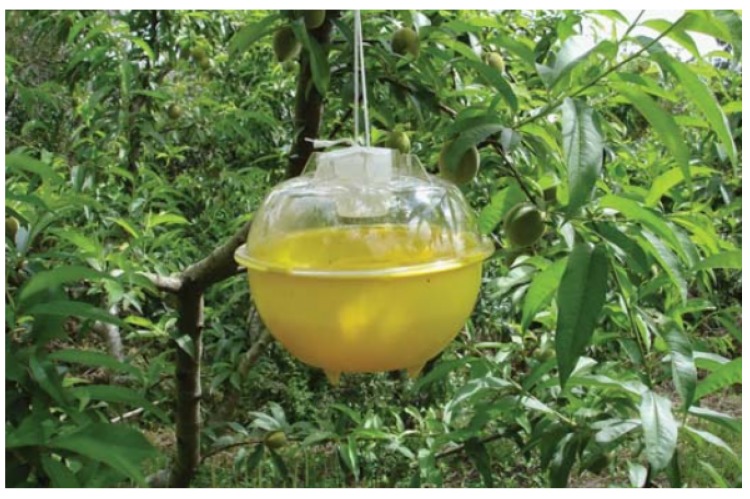
McPhail trap during peach orchard monitoring.

For the management of fruit flies, monitoring is essential through the use of attractive bait traps. One of the possible types of traps is McPhail that uses food attractions to catch adult fruit flies (Figure 2). It is also possible to use sex pheromones to attract adults, such as the paraferomonium used in Deltas traps to attract *C. capitata*. When the traps are used for monitoring, a technician must perform the inspection of the traps by classifying and counting the captured flies.

For the use of an intelligent monitoring system that automatically counts and identifies fruit flies, replacing the use of a specialized technician for this task will both minimize human errors in the identification and counting of flies and reduce the cost of the monitoring program. The system can be used both as auxiliary means to corroborate the survey carried out by the technician, as it may be the main means in situations that there are difficulties for the presence of the technician.

The use of optoelectronics for insect identification was presented by [5], where a photoreceptor was used to capture the variation of ambient light generated by the beating of the insect wings during flight. This variation of light is processed and determines the frequency of the beating of the insect wings. According to [6], this frequency depends on the physiological characteristics of the species and can be used for its identification.

Currently, this technique has been employed in the development of an intelligent mosquito trap [7,8,9]. For these works, a low cost optoelectronic sensor has been developed in which a light barrier is created with the use of a low power laser. The created light is reflected by a reflector being picked up by a phototransistor placed next to the laser. Thus, when the insect crosses the light barrier, it is partially occluded by its wing movement, this variation being captured by the phototransistor [7].

Optoelectronic sensors were also used in the design of the intelligent trap for olive fruit flies *Bactrocera oleae* (Rossi, 1790) [10,11,12,13,14]. The developed sensor uses the infrared LED (LED-IR) as an emitter to create the light barrier and as receivers uses photodiodes.

For the extraction of the characteristics of the signal, the analysis of the frequency spectrum is used to locate the fundamental frequency of the signal [7], frequency spectrum, circadian rhythm of flight activity and geographical location [8], Mel-frequency cepstral coefficients (MFCC) [9], use of the root mean square value (RMS) of the captured signal to detect passing events [10], use of Fast Fourier transform(FFT) with Hamming windowing to obtain the captured signal spectrum [11] and FFT to obtain the fundamental frequency, differences between the real harmonics and the theoretical harmonics, the distribution of energy in the harmonics and the amplitude of the signal near the frequency of 0 Hz (related to the body size of the insect) [14].

Several types of classifiers are used for the identification of insects through the wing beat signal such as: Bayesian classifiers [7,8], Support Vector Machines (SVM) with Radial Basis Function (RBF) [9], comparison with a reference spectrum using the algorithm K-means [11], and classification through a set of rules when using traps with attractive pheromone and without the presence of other fruit flies. In the work presented by [14], several classifiers such as Linear Support Vector Machines, Radial Basis Function kernel Support Vector Machine [14], Random Forests, Adaptive Boost, X-TREE, Group-Based Classification and Convolutional Neural Network were analyzed.

This work presents the development of an optoelectronic sensor for use in a McPhail trap and the study of signal processing techniques for detecting and discriminating fruit flies (*C. capitata* and *A. fraterculus*) in real time, which can be integrated into an automated alert system to inform farmers about pest status. A desirable feature of the sensor was to use readily available and inexpensive parts. A dataset of the signals generated by wing beat of flies was produced for this work and made available for later performance evaluation.

## 2. Materials and Methods

### 2.1. Optoelectronic Sensor Prototype

**Figure 3:**
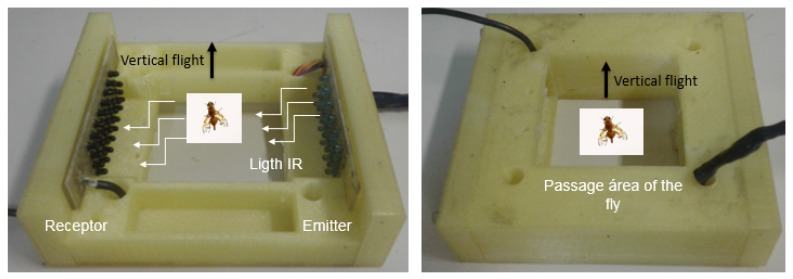
Optoelectronic sensor base with emitter and receiver circuits (**left**) and optoelectronic sensor ready for use (**right**).

**Figure 4:**
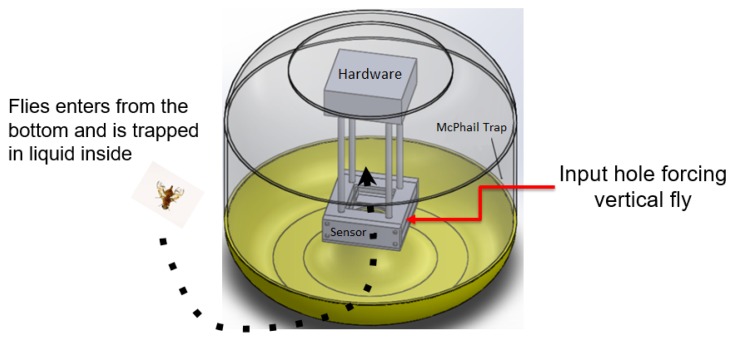
Proposal of a McPhail intelligent trap using the developed sensor optoelectronic sensor.

The developed optoelectronic sensor was based in the work presented by [15], where the authors present an optoelectronic sensor using as emitters infrared LEDs and, as phototransistors receivers, the hardware was developed for the treatment of the captured signal and a base for the sensor to be coupled to a McPhail trap. The sensor was evaluated through an insect wing beat simulator developed for the work. The base developed with the emitter and receiver circuits is shown in Figure 3, the infrared LED TIL32 being used as an emitter and the phototransistor TIL78 as a receiver. The drawing of the fly in Figure 3 indicates the area of passage through the sensor and the direction of flight of the flies. Thus, the sensor may be coupled to a McPhail trap for the future development of an intelligent trap (Figure 4).

**Figure 5:**
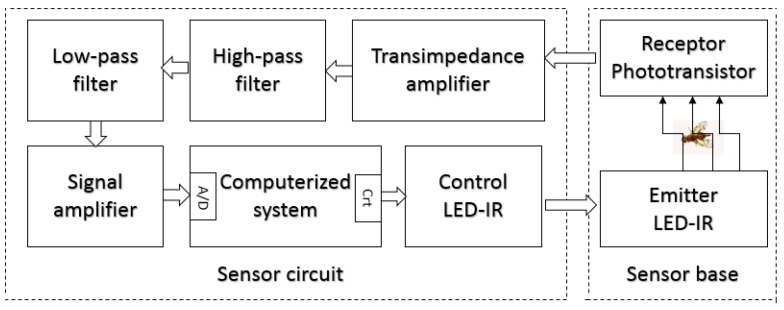
Diagram in sensor circuit blocks.

**Figure 6:**
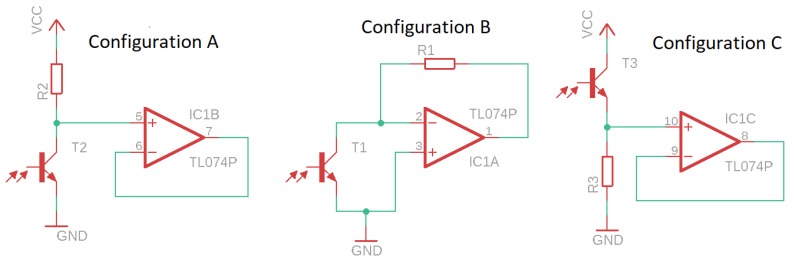
Evaluated configurations for the transimpedance circuit.

**Figure 7:**
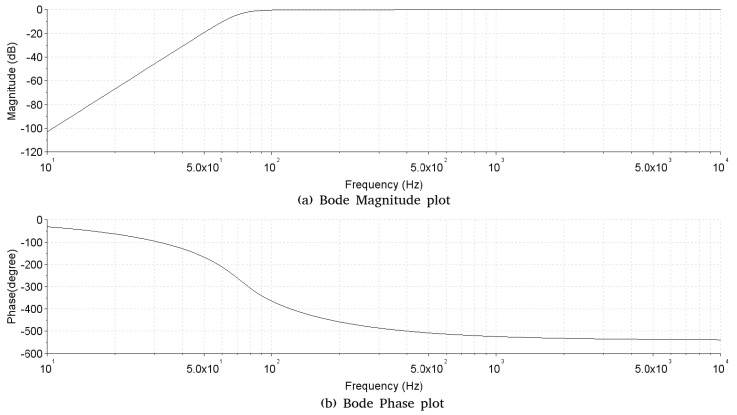
Bode diagram, with the Bode Magnitude plot (**a**) and the Bode Phase plot (**b**), of analog high-pass filter implemented.

**Figure 8:**
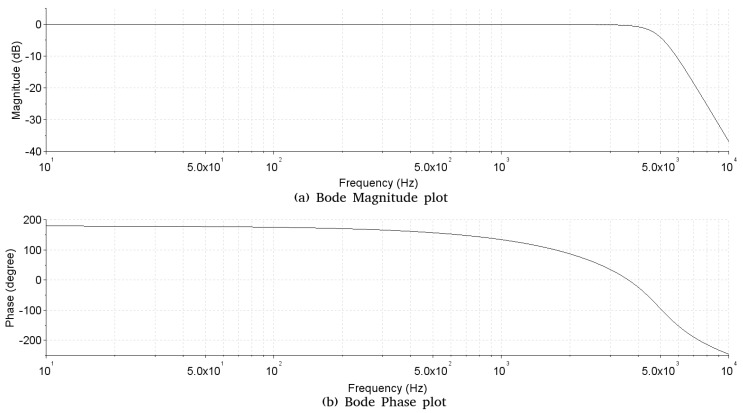
Bode diagram, with the Bode Magnitude plot (a) and the Bode Phase plot (b), of analog of analog low-pass filter implemented.

The hardware for the optoelectronic sensor proposed by [15] was revisited and modified, being divided into eight functional blocks based on the diagram presented in Figure 5. The blocks LED-IR, phototransistor receiver and LED-IR control were not modified and the blocks transimpedance amplifier, analog high-pass filter, analog low-pass filter, amplifier signals and computerized system were modified, being described in the sequence:

1. **Transimpedance amplifier**—The block of the transimpedance amplifier has the function of converting the current generated by the incidence of light on the phototransistors into a voltage. For the circuit of the transimpedance amplifier, three configurations were evaluated (Figure 6), the one that presented better performance in the experiments with respect to the noise and signal distortion was configuration A.
2. **Analog high-pass filter**—The analog high-pass filter has as its function the elimination at the DC level generated by the base light emitted by the TIL32, allowing the passage of only the electrical signal corresponding to the variation of light caused by the beating of the wings of the insects. The Butterworth approximation was used to calculate the filter, since it has a flat frequency response in the passband without the presence of ripple at the cut-off frequency. This characteristic is important because of the close-up of the cut-off frequency of the high-pass filter (70 Hz) with the lowest frequency of interest 89.1 ± 4.5 Hz (fundamental frequency of the wing beat signal of the *A. fraterculus* [6]). The order of the implemented filter was determined experimentally by evaluating the noise generated by the electric network (60 Hz) that is close to the cut-off frequency (70 Hz). Thus, a sixth order filter was implemented using the Butterworth approximation, having as characteristics a cut-off frequency of 70 Hz, unit gain in the bandwidth, attenuation of −60 dB per decade and a 0 dB ripple in the passband. The bode diagram of analog filter implemented is shown in Figure 7, where the cut-off frequency is observed at 70 Hz without ripple and with an attenuation of −10 dB at 60 Hz. For the filter design, the Multiple Feedback topology (MFB) was used. The filter was implemented with three second order stages connected in cascade. The choice of the MFB topology was due to its greater robustness to the variation of the component values [16], allowing the use of components with higher tolerance and lower cost.
3. **Analog low-pass filter**—The analog low-pass filter has the function of limiting the upper frequency of the passband, serving as an anti-alias filter. Thus, the use of a low-pass filter of sixth order, cut-off frequency of 5000 Hz, with unit gain in the passband and 0 dB ripple in the passband was defined. In this way, the filter allows the passage of the frequencies of interest that are below 1000 Hz and that in the future a sample rate of at least 10 ksamples/s could be used, without modifications in the hardware. Using the Butterworth approximation and the design requirements, the following transfer function was obtained for the low pass filter. The bode diagram of the filter developed using the Butterworth approximation is shown in Figure 8, where the cut-off frequency is observed at 5000 Hz without ripple. The low-pass filter design was performed using the MFB topology with a three-stage implementation of second order cascade connected.
4. **Signal amplifier**—The signal amplifier block functions to amplify the signal conditioned by the analog filters on the full scale of the A/D converter. In this way, it is possible to obtain a higher resolution of the signal during the conversion. This block is composed of a two-stage amplifier, and both stages were designed using an operational amplifier configured as an inverter amplifier. The two-stage configuration was chosen to minimize the signal offset error caused by a high gain single stage amplifier. To determine the total gain required in the signal amplifier, we considered: the full scale voltage of 2 Vpp of the line input of the sound card used as the A/D converter, the intensity of the light generated by the transmitter with an active and current line of polarization of 20 mA and gain adjustment was made using as reference the passage of *A. fraterculus*, which, being the largest insect, caused the greatest variation in signal amplitude. Thus, the signal amplifier circuit has a total gain of 270 with a lower cut-off frequency of 0.72 Hz and a cut-off frequency over 48.2 kHz. The sensor hardware composed of the transimpedance amplifier, analog high-pass filter, analog low-pass filter and signal amplifier has an estimated total gain of $405\times 10^{6}$, lower cut-off frequency of 70 Hz, and a higher cut-off frequency 5 kHz.
5. **Computerized system**—In the implementation of the computerized system block, a Dell Optiflex 9010 PC computer was used, with an Intel (R) Core i5-3570M 3.40 GHz processor, 8.00 GB memory, Windows 10 PRO operating system, sound Realtek High Definition Audio version 6.0.1.6075. Thus, the output of the signal amplifier was connected to the audio line input of the computer, using the A/D converter of the audio card for the conversion of the captured signal. Audacity software version 2.1.3 was used to record signal files. The amplitude of the recorded signal was normalized by the software to values between −1 and 1. The recording was performed with 16-bit resolution, mono and sampling rate of 192 ksamples/s. Although the sensor has an upper cut-off frequency of 5 kHz, allowing a minimum sampling rate of 10 ksamples/s, the highest sampling rate available in the computerized system (192 ksamples/s) was used. This allowed, at this stage of the project, to verify in the captured signal the existence of undesired frequencies generated by spurious sources (e.g., computerized system or due to problems in the design of the sensor), which could hinder signal analysis. Another goal was to make the dataset more flexible, allowing the future evaluation of lower sampling rates, by performing sub-sampling of the signals that make up the dataset. The digital processing of the captured audio signals was performed using SciLab software version 6.0.0, where the scripts used in the analysis were implemented.

### 2.2. Method of Measuring the Signals Generated by the Beating of Wings of Fruit Flies *A. fraterculus* and *C. capitata*

The Embrapa Clima Temperado company, located in the city of Pelotas, RS, Brazil, provided the *A. fraterculus* and *C. capitata* flies used in the experiments. The flies were produced in the laboratory according to the creative technique [17]. Thus, insects with integral wings and with full flight capacity were used.

**Figure 9:**
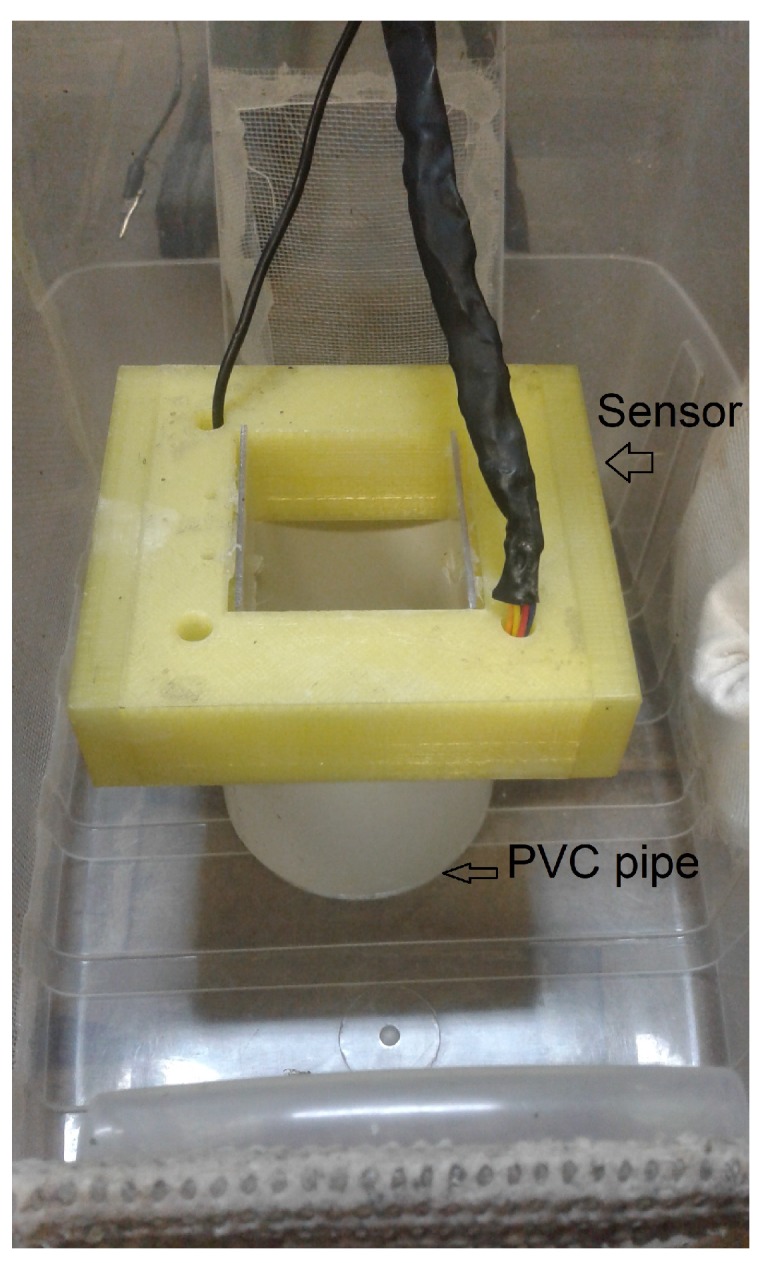
System used in the experiment to measure the signal of fruit flies, with the detail of the positioning of the PVC pipe and the sensor.

The system used in the experiment to measure the wing beat signal generated by *A. fraterculus* and *C. capitata* (Figure 9) consisted of an adapted cage where puparium was placed within a PVC pipe (10 cm length × 10 cm diameter), positioned under the sensor passage area. The interior of the PVC tube was coated with white talc to prevent the flies from climbing walking. Forcing them to perform a vertical flight upwards to leave the PVC pipe. Thus, flying through the passage area of the sensor. The experiments were performed with natural light and with an ambient temperature of 28 ± 1 ${}^{\circ }$C.

Signal recording was performed by connecting the output of the sensor signal amplifier to the line input of the computer system sound card. The signal was recorded using the Audacity software that normalized the amplitude of the signals to values between −1 and 1. Recording was performed on signal tracks for one hour each. The experiment was completed when it was visibly observed that the remaining insects did not make attempts to fly out of the PVC pipe passing through the sensor.

**Figure 10:**
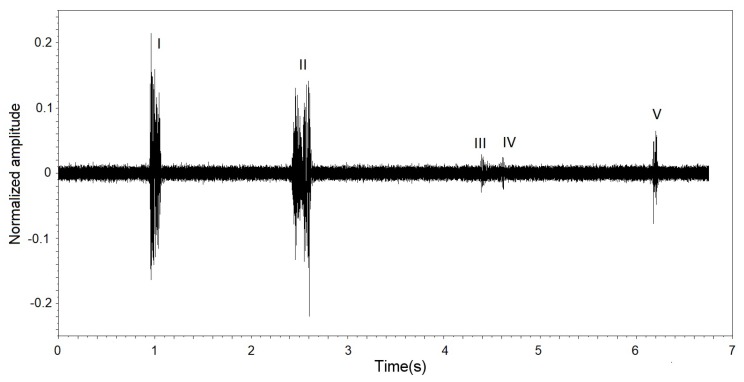
Signal extract captured with brightness fluctuations in I, II, III, IV and V.

For the identification of possible events of passage of flies, the algorithm of detection of events of passage (PEDA) was developed. The algorithm calculates the RMS value of the captured signal and compares it to an experimentally established detection threshold. A passing event is detected when the RMS value exceeds the detection threshold. The RMS value was obtained using Equation (1), with a sliding window of 30 ms on the signal and 10 ms overlap between the windows:

(1) $$RMS\;value=\sqrt{\frac{1}{n}\sum_{i=1}^{n}x_{i}^{2}},$$

where *n* represents the number of samples in the evaluation window. When the RMS value exceeds an established threshold value, it was considered that a possible event of passage of a fly occurred through the sensor. Figure 10 presents a captured signal extract, with background noise and luminosity fluctuations (I, II, III, IV and V).

**Figure 11:**
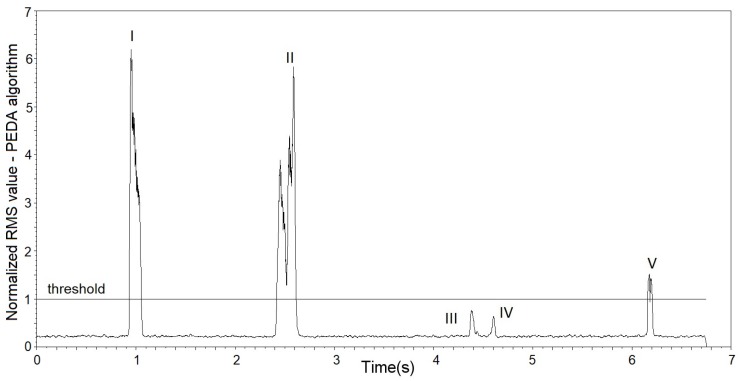
Results of the RMS value using threshold one obtained with the PEDA algorithm applied to the signal of Figure 10, with passages detected in I, II and V and disregarded in III and IV.

Figure 11 shows the result of the PEDA algorithm with the threshold of 1 applied to the signal shown in Figure 10. It can be observed that insect passage events exceeded the considered threshold (I, II and V) and insect fluctuations that tried to pass through the sensor and did not obtain success being below the threshold considered (III and IV).

**Figure 12:**
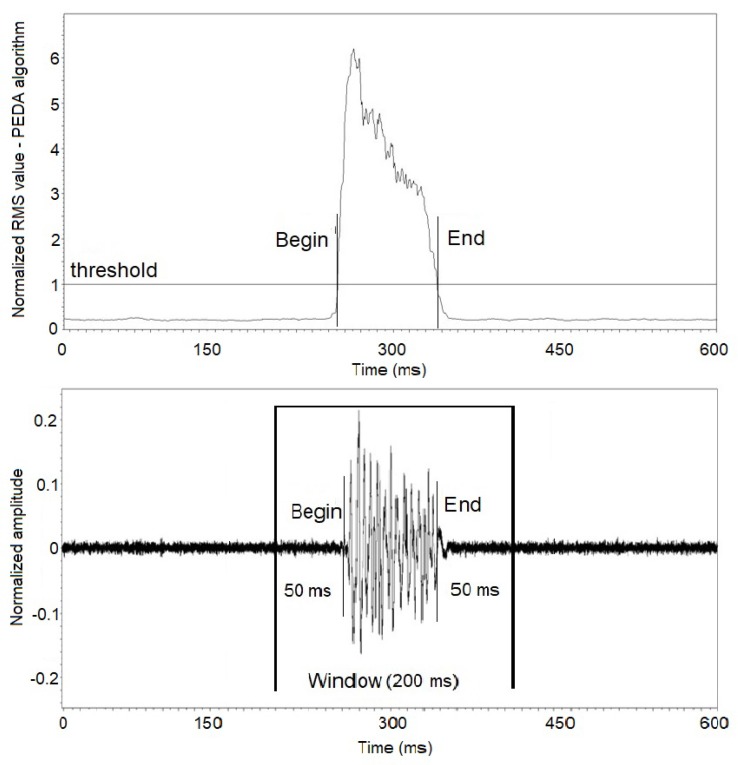
Stored signal window based on insect passage detection.

When a passing event is detected, its start and end are evaluated and, based on these values, the signal window of the event is stored. The window was stored with the 50 ms of signal before the start of the event and with the 50 ms of signal after the end of event (Figure 12). This interval has been defined experimentally to ensure that the signal from the insect passage is within the window.

The localized events were analyzed and classified into a standard group for characterization of the insect signal. The criterion used to classify events in the standard group was that the signal should be at least 100 ms in duration without considering the 50 ms added at the beginning and end of each passing event and be a direct passage through the sensor.

For the extraction of characteristics of the signals of detected events of passage, an algorithm of automatic extraction of characteristics (CAEA) was developed. Initially, a Blackman–Harris window was applied to the stored signal, as suggested by [18], with its mathematical model given by

(2) $$\omega \left(n\right)=a_{0}-a_{1}cos\left(\frac{2\pi n}{N-1}\right)+a_{2}cos\left(\frac{4\pi n}{N-1}\right)-a_{3}cos\left(\frac{6\pi n}{N-1}\right),$$

where $a_{0}=0.35875$, $a_{1}=0.48829$, $a_{2}=0.14128$ and $a_{3}=0.01168$.

The application of the Blackman–Harris window on the stored signal consists of calculating the mathematical model of the window used for each stored point of the signal; this allows for the smoothing of the edges caused by the clipping of the event signal of the original signal, minimizing the appearance of frequencies nonexistent or fictitious in the spectrum of this signal during the FFT. With the signal submitted to the Blackman–Harris window, it has been expanded by inserting new samples with a value of zero until the size of the analysis window has the time of one second. In this way, a resolution of 1 Hz was obtained in the execution of the FFT method (CAEA algorithm), allowing a more detailed analysis of the wing beat signal of the insects.

The CAEA algorithm uses the autocorrelation and FFT techniques to extract the characteristics of the signal. The autocorrelation (Equation (3)) was used to obtain information on the fundamental frequency of signal generated by the wing beat of flies. The autocorrelation method was implemented using the xcorr() function of Scilab:

(3) $$r_{xx}\left(l\right)=\sum_{i=1}^{Nw}x\left(i+l\right)x\left(i\right).$$

**Figure 13:**
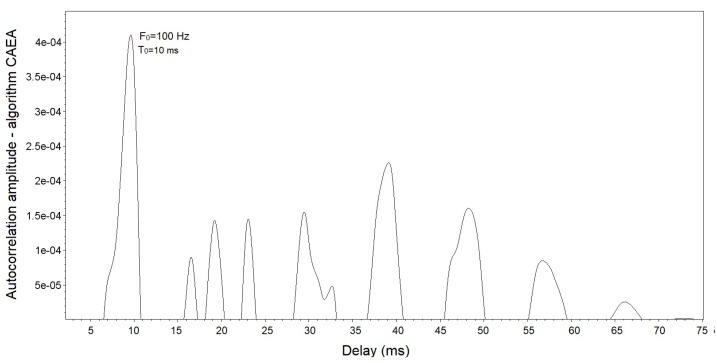
Results of the CAEA algorithm (autocorrelation method) with a fundamental frequency in 100 Hz.

The results of the CAEA algorithm (autocorrelation method) for the signal shown in Figure 12 are presented in Figure 13, where it is noted that the peak of greater amplitude has a delay of 10 ms. This means that the analyzed signal has a fundamental period (T0) of 10 ms, that is, a fundamental frequency (F0) of 100 Hz.

For the analysis of the frequency spectrum of the signal, the CAEA algorithm uses the FFT method Equation (4), being implemented with the function fft() of Scilab. The peak of greater magnitude is located in the frequency spectrum of the signal, considered the fundamental frequency of the signal. After the next four peaks of greater magnitude are located, they are located above the fundamental frequency, considering the other four analyzed components of the signal:

(4) $$FFT=\sum_{i=0}^{W_{n}-1}x_{i}.e^{\frac{-j2\pi }{W_{n}}}.$$

**Figure 14:**
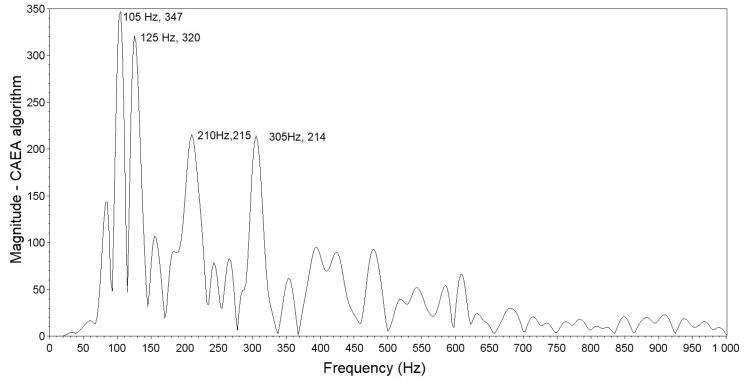
Frequency spectrum of the signal obtained by the CAEA algorithm (FFT method).

The frequency spectrum result obtained with the CAEA algorithm (FFT method) in the signal analysis of Figure 12 is presented in Figure 14. Note that the fundamental frequency of 105 Hz corresponds to that obtained by the CAEA algorithm (autocorrelation method) (100 Hz) and the peaks of magnitude at 125 Hz, 210 Hz and 305 Hz correspond to the other four frequency components.

The characteristics of the signal of each passing event obtained with the CAEA algorithm were stored in a file and the statistical analysis of the data was performed. The statistical methods applied were based on the one proposed by [19], using software [20] for their implementation.

For the analyses, we obtained the descriptive measures statistical arithmetic mean ($\bar{X}$), sample standard error ($S_{x}$), sample standard deviation (*S*), kurtosis coefficient (*K*), Coefficient of asymmetry (As), sample range (*H*), minimum sample value (Min) and maximum sample value (Max).

Regarding the frequency data, we analyzed the fundamental frequency by the CAEA algorithm (autocorrelation method), the five frequency components by the CAEA algorithm (FFT method) and the differences between the fundamental frequency by the CAEA algorithm (FFT method) and the frequencies of the other four components by the CAEA algorithm (FFT method). In the magnitude data, we analyzed the magnitudes of the five frequency components obtained by the CAEA algorithm (FFT method), the relations between the magnitude of the fundamental frequency by the magnitudes of the other four components and the relationships among the magnitudes of the subsequent components.

In order to verify the normality of the statistical analysis, the one proposed by [21] was used, and the graphical evaluation was performed using boxplot, frequency distribution (histogram) and P–P (probability–probability). As a complementary evaluation to the graphical evaluation of normality, the Shapiro–Wilk test was performed, where a *p*-value > 0.05 was obtained as a response and the distribution can be considered normal.

In the data that can be represented by a normal distribution and, due to the size of the samples, the T-Student distribution was used to obtain the confidence interval of the population mean, being considered a confidence level of 95%. Thus, the confidence interval of the population mean is given by

(5) $$IC\left(\mu \right)=\bar{X}\pm T.S_{x},$$

where IC(μ) is the population mean with the confidence interval, $\bar{X}$ is the sample mean, $S_{x}$ is the standard error and T is the correction value obtained by the distribution T-Student.

To evaluate the probability of identification errors among fruit flies using the characteristics extracted from the captured wing beat signal, the cumulative probability value was used for a normal distribution, Equation (6), based on the intersection of the curves of the evaluated characteristics of the insects:

(6) $$Pr\left(x_{1}\leq x\leq x_{2}\right)=\int_{x_{1}}^{x_{2}}\frac{1}{\sqrt{2\pi \sigma^{2}}}e^{-\frac{{\left(x-\mu \right)}^{2}}{2\sigma^{2}}}dx,$$

where $x_{1}$ and $x_{2}$ represent the cumulative probability interval, *x* the integrated sample set, σ the population standard deviation, and μ the population mean.

## 3. Results and Discussion

### 3.1. Measurement of the Wing Beat Signal Generated by A. fraterculus

**Figure 15:**
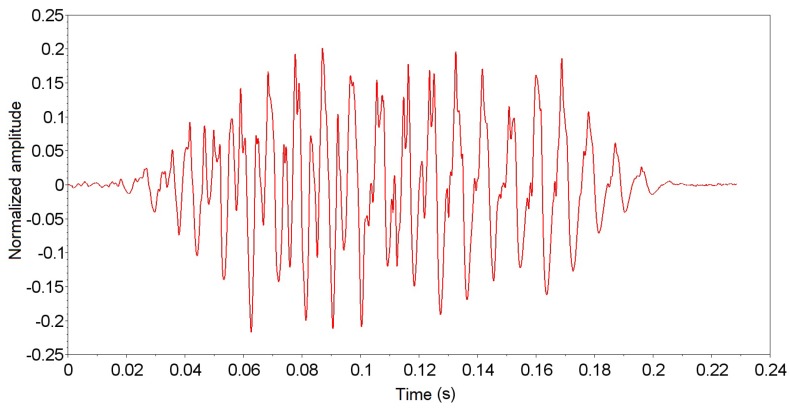
*Anastrepha fraterculus* passing event signal classified in the standard group.

**Figure 16:**
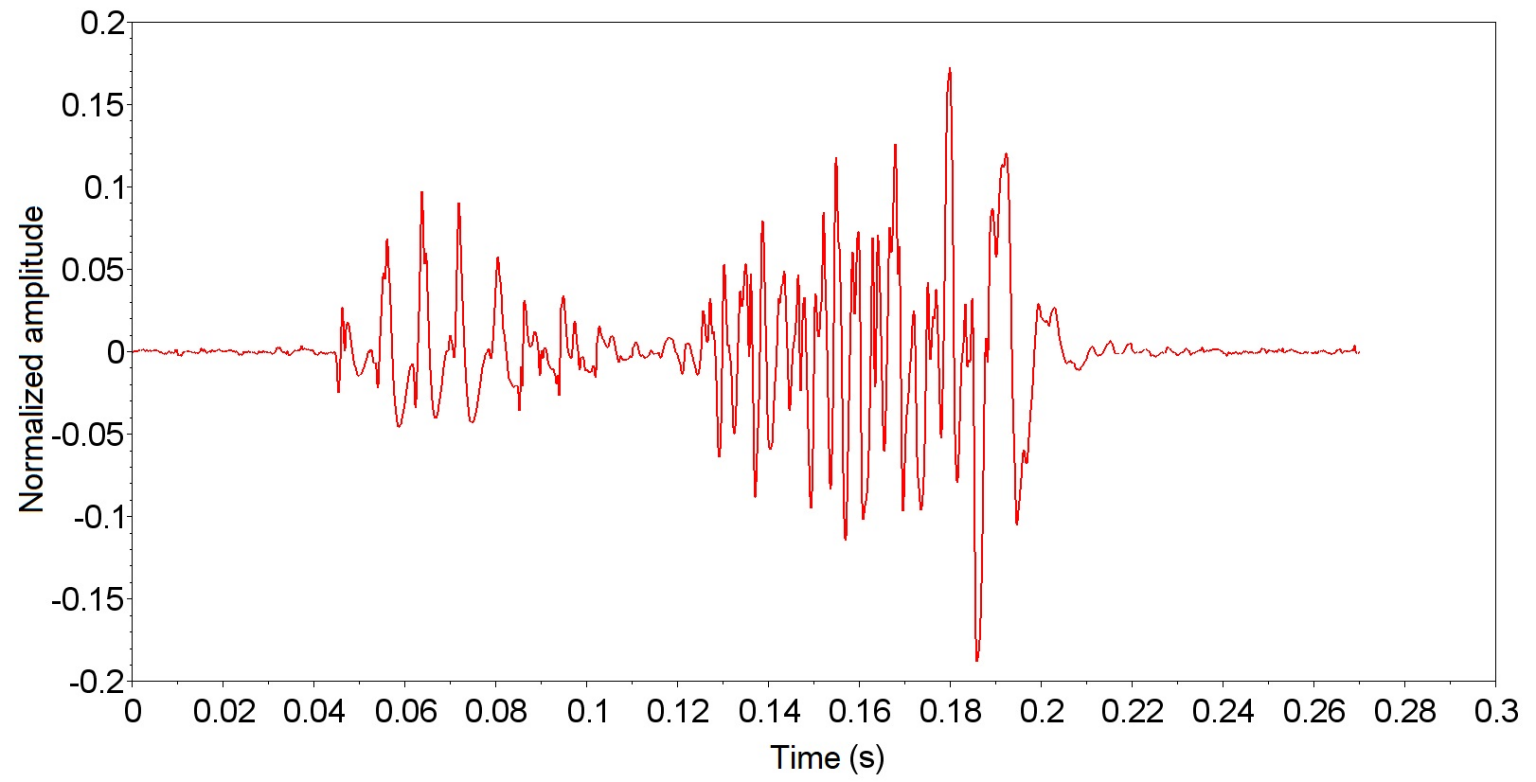
*Anastrepha fraterculus* passing event signal not classified in the standard group.

The experiments performed with *A. fraterculus* were recorded at about seventeen hours of signal, separated into seventeen signal tracks of one hour each, for easier signal processing software. Each signal track was submitted to the PEDA algorithm, with 466 possible events of passage. The possible localized passage events were analyzed and classified in the standard group for the characterization of the wing beat signal generated by *A. fraterculus*. The localized events were analyzed and classified into a standard group for characterization of the insect signal, and 66 events were selected from the 466 events located (according to the criteria presented in Section 2.2). Figure 15 displays an event passing signal of *A. fraterculus* sorted in the default group, and Figure 16 displays an unordered event signal in the default group. The unclassified event signal in the standard group was discarded for having a decrease in signal strength (time 0.12 s), despite having a duration greater than 100 ms. This decrease in signal intensity represents that the insect did not make a direct passage through the sensor.

**Figure 17:**
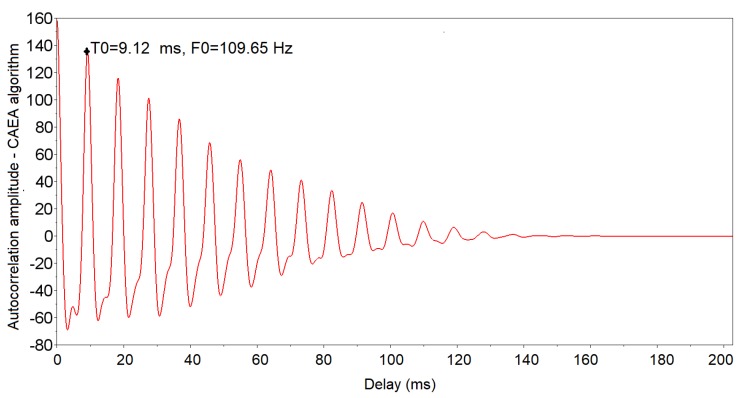
Results of the CAEA algorithm (autocorrelation method) graph of the *A. fraterculus* passing event of the standard group.

The 66 events of the standard group were analyzed and the characteristics of the signals were extracted using the CAEA algorithm. Figure 17 shows the output resulting from the CAEA algorithm (autocorrelation method) of one of these events. Note that the CAEA algorithm output highlights the fundamental period of the signal (peak of greater amplitude), the calculation of its inverse to obtain the fundamental frequency being necessary. Thus, the fundamental period (T0) of 9.12 ms corresponding to the fundamental frequency (F0) of 109.65 Hz was obtained.

**Figure 18:**
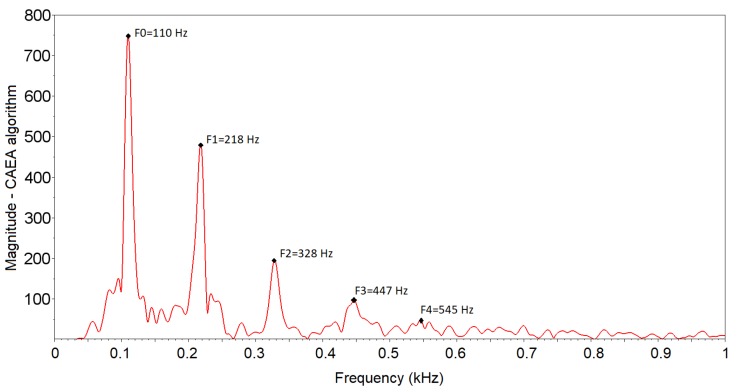
Frequency spectrum of the signal of an event of passage of the standard group of *A. fraterculus* obtained by the CAEA algorithm (FFT method).

At the output of the CAEA algorithm (FFT method), the five highest peaks corresponding to the fundamental frequency (F0), 2nd component (F1), 3rd component (F2), 4th component (F3) and 5th component (F4), respectively, were detected Figure 18. The values obtained with the algorithm are 110 Hz (fundamental frequency), 218 Hz (2nd component), 328 Hz (3rd component), 447 Hz (4th component) and 545 Hz (5th component). It was observed that, for the 4th component and 5th component, a degradation of the signal occurs that makes it difficult to correctly detect the corresponding peaks. This degradation of the signal occurs due to the use of the phototransistor as a receiver element, as already observed by [11,15].

The data obtained by extracting the characteristics of the signals from the 66 events of passage of the standard group of *A. fraterculus* stored in the file were analyzed and the descriptive measures’ complete statistics are presented in Appendix A. Table A1 and Table A2 present the descriptive measures of the data of the complete samples. In addition, Table A3 and Table A4 present the descriptive measures with the removal of outliers in each characteristic of the analyzed signal, and we considered data outliers that are outside the minimum and maximum limits of the boxplot.

**Figure 19:**
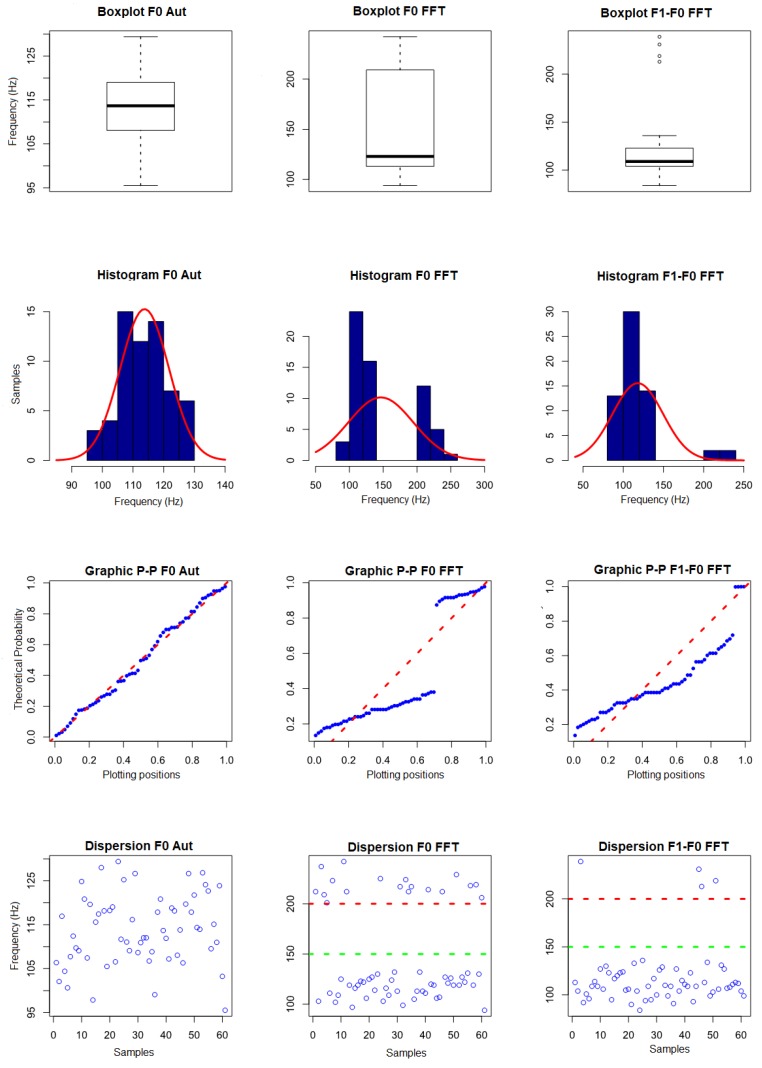
Graphics of the visual evaluation of the data obtained with the events of passage of *A. fraterculus* of the standard group without removal of outliers.

In the analysis of the complete data without removal of outliers (Table A1), it was observed that, due to the degradation in the higher frequency frequencies obtained from the signals, as previously reported, there was a difficulty in locating the peaks corresponding to the their components. Thus, it was not possible to use the data for characterization of the signal, since they present great variation between the minimum and maximum values. Thus, the fundamental frequency obtained by the CAEA algorithm (autocorrelation method) (F0 Aut), the fundamental frequency obtained by the CAEA algorithm (FFT method) (F0 FFT) and the frequency obtained by the difference between the frequency of the 2nd componte and the fundamental frequency, both obtained by the CAEA algorithm (FFT method) (F1-F0 Aut), were analyzed. Figure 19 presents the visual evaluation performed.

In the first line of Figure 19, the boxplot graphics are presented, where, in the measurement of the fundamental frequency by autocorrelation, a symmetrical distribution is observed with the midline in the center of the box, with symmetrical mustaches and slightly longer than the subsections of the box and no outliers data, indicating a normal distribution. In the data concerning the measurement of the fundamental frequency obtained in the CAEA algorithm (FFT method), we observe an asymmetric distribution with the median line near the lower part of the box, asymmetric and short mustaches, a sparse distribution pattern in relation to the medium generating a long box, not presenting data outliers, indicating a distribution that may not be represented by a normal one. In the data concerning the frequency of the difference between the 2nd component and the fundamental frequency obtained in the CAEA algorithm (FFT method), an asymmetrical distribution is observed with the median line near the lower part of the box, slightly asymmetrical mustaches and slightly longer than the subsections of the box and four outliers above 200 Hz, indicating that this distribution can be represented by normal distribution.

In the second line of Figure 19, the histograms (blue) and curve of the superimposed normal (red) distribution are presented. With respect to the data of the first column, it is observed the highest concentration of values near the mean, the distribution being practically symmetrical, with the format of bell, without gaps in the data and without outliers data, indicating a normal distribution. In the second column, we observe the displaced mean being to the right of most of the data, presenting a gap between the data and the left of the mean of a new concentration of data, indicating a non-normal distribution. In the third column, we observe a greater concentration of data slightly to the left of the mean, having some data outliers above 200 Hz, indicating a normal distribution.

In the third line of Figure 19, we present the P–P graphics with the probability distribution of the sample data (blue) superimposed on the probability distribution of a normal curve (red). In the first column, it is observed that the probability distribution of the data tends to follow the probability distribution of a normal curve, indicating a normal distribution. In the second column, it is observed that the probability distribution of the data does not follow the distribution of probability of a normal curve, with a gap between the data, indicating a non-normal distribution. In the third column, it is observed that the probability distribution of the data does not follow the distribution of probability of a normal curve, with a gap and downward spacing caused by the outliers data, indicating a non-normal distribution.

As a complementary evaluation, the normality test of Shapiro–Wilk was performed, obtaining a *p*-value of 0.7018 (above the limit of 0.05 for normality) for fundamental frequency by the CAEA algorithm (autocorrelation method), *p*-value of $1.589\times 10^{-8}$ (below the 0.05 limit for normality) for the fundamental frequency obtained in the CAEA algorithm (FFT method) and a *p*-value of $9.097\times 10^{-11}$ (below the limit of 0.05 for normality) for the frequency of the difference between the 2nd component and the fundamental frequency obtained in the CAEA algorithm (FFT method). Based on the visual evaluation and the Shapiro–Wilk normality test, it was found that the fundamental frequency data obtained by the CAEA algorithm (autocorrelation method) can be represented by a normal distribution, the fundamental frequency data obtained by the peak location in the CAEA algorithm (FFT method) can not be represented by a normal distribution and that the frequency data obtained by the difference between the frequency of the 2nd component and the fundamental frequency both obtained by the location of its peaks in the CAEA algorithm (FFT method) can not be represented by the normal distribution.

**Figure 20:**
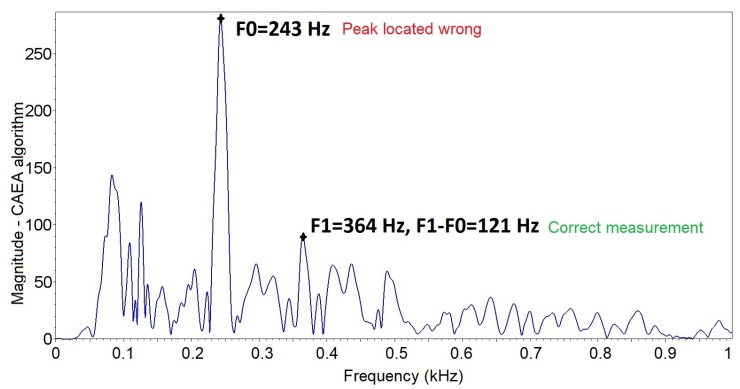
Frequency spectrum obtained by the CAEA algorithm (FFT method) with peak location error referring to the fundamental frequency of *A. fraterculus* of the standard group.

**Figure 21:**
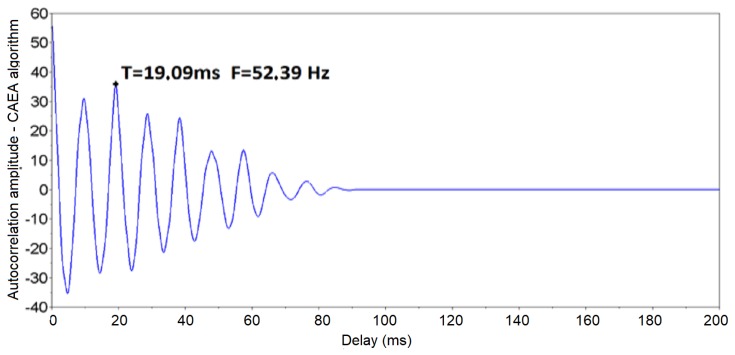
The CAEA algorithm (autocorrelation method) graph with peak location error referring to the fundamental frequency of *A. fraterculus* of the standard group.

With the results obtained with the normality evaluation, the visual analysis of the data dispersion (fourth line—Figure 19) was performed, being observed in the third column that the data are grouped below 150 Hz (green line), with 62 occurrences and above 200 Hz (red line), with four occurrences, these data being considered outliers that can be removed from the dataset. In the second column, the data are grouped below 150 Hz (green line), with 48 occurrences and above 200 Hz (red line), with 18 occurrences. Based on the performed analyses, it is noted that data above 200 Hz, although not considered outliers, represent that the localized peak does not correspond to the fundamental frequency of *A. fraterculus* (Figure 20)—it being possible to remove them from the dataset. The fundamental frequency obtained by the CAEA algorithm (autocorrelation method) presented errors of evaluation (Figure 21), in which case the measured value is below the expected one.

**Figure 22:**
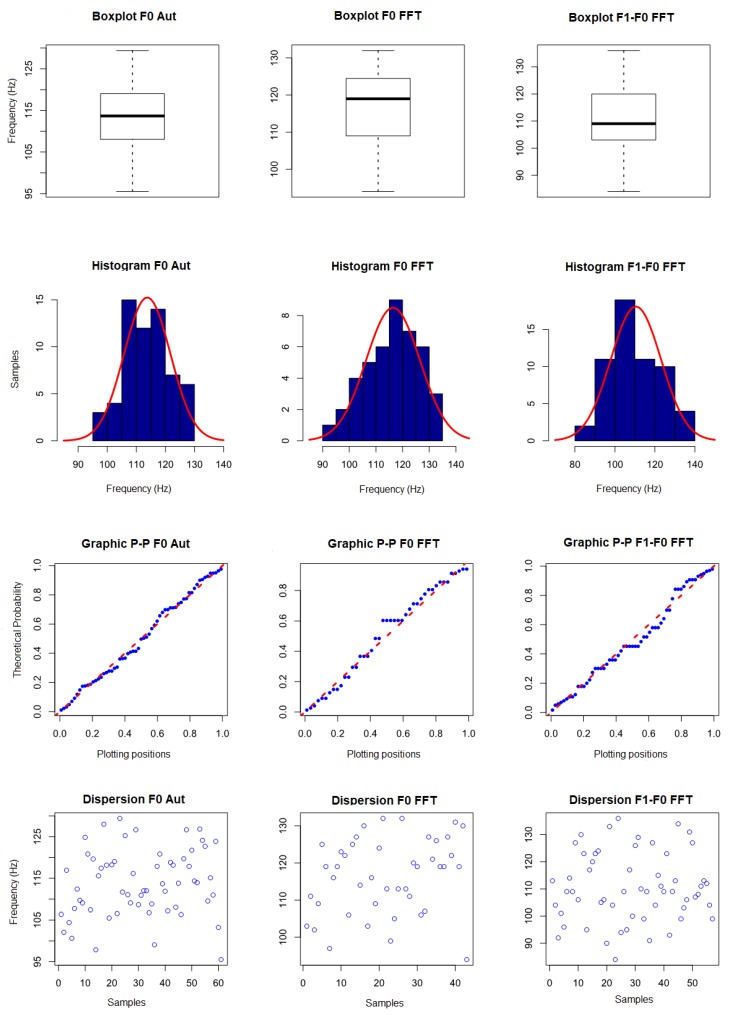
Graphics of the visual evaluation of the data obtained with the events of passage of *A. fraterculus* of the standard group with removal of outliers.

Figure 22 presents the visual evaluation of the data with removal of outliers referring to Table A3. Each column, from left to right, presents the data for fundamental frequency measurements obtained by the CAEA algorithm (autocorrelation method), fundamental frequency obtained by the location of the peak in the CAEA algorithm (FFT method) and the frequency obtained by the difference between the frequency of the 2nd component and the fundamental frequency both obtained by the location of their peaks in the CAEA algorithm (FFT method). The results for the fundamental frequency obtained by the CAEA algorithm (autocorrelation method) were not altered, once they did not have outliers data, kept for comparison with the data obtained with the removal of the outliers of the fundamental frequency obtained in the CAEA algorithm (FFT method) and the frequency by the difference between the 2nd component and the fundamental frequency obtained in the CAEA algorithm (FFT method).

In the first line of Figure 22, the boxplot graphics are displayed. In the data concerning the measurement of the fundamental frequency obtained in the CAEA algorithm (FFT method), second column, a slightly asymmetrical distribution is observed with the median line near the upper part of the box, slightly asymmetrical mustaches and no outliers data, indicating that this distribution may be normal. In the data concerning the frequency of the difference between the 2nd component and the fundamental frequency obtained in the CAEA algorithm (FFT method), third column, a slightly asymmetrical distribution is observed with the median line near the lower part of the box, slightly asymmetrical mustaches and slightly longer than the subsections of the box and without outliers, indicating that this distribution may be normal. In relation to the fundamental frequency boxplot by the CAEA algorithm (autocorrelation method), the first column, the graph with the greatest similarity is the one in the third column, and the one in the second column presents a larger dispersion data and with a larger average.

In the second line of Figure 22, the histograms (blue) and curve of the superimposed normal distribution (red) are shown. In the second column, we observe the highest concentration of values close to the average with a longer tail on the left, with the bell format, with no data gaps and no outliers data, indicating a normal distribution. In the third column, we observe the highest concentration of near-average values with a longer tail on the right, with the bell shape, without gaps in the data and without outliers data, indicating a normal distribution. In comparison with the histogram of the first column, the similarity between its distributions is observed, being that in the second and third columns the distribution is more sparse.

In the third line of Figure 22, we present the P–P graphics with the probability distribution of the sample data (blue) superimposed on the probability distribution of a normal curve (red). In the second and third columns it is observed that the probability distribution of the data tends to follow the probability distribution of a normal curve, indicating that both can be represented by a normal distribution.

The Shapiro–Wilk normality test was performed as a complementary assessment, obtaining a *p*-value of 0.2211 (above the limit of 0.05 for normality) for the fundamental frequency obtained by the CAEA algorithm (FFT method) and a *p*-value of 0.287 (above the limit of 0.05 for normality) for the frequency of the difference between the 2nd component and the fundamental frequency obtained in the CAEA algorithm (FFT method). Based on the visual evaluation and the Shapiro–Wilk normality test it was observed that the fundamental frequency data obtained by the peak location in the CAEA algorithm (FFT method), considering the erroneous detections as outliers, can be represented by a normal distribution and the frequency data obtained by the difference between the frequency of the 2nd component and the fundamental frequency both obtained by the location of its peaks in the CAEA algorithm (FFT method), without the outliers data, can be represented by the normal distribution.

Due to sample sizes (66 for fundamental frequency by the CAEA algorithm (autocorrelation method), 46 for fundamental frequency by FFT and 62 for frequency by difference between peaks in FFT) and that the data can be represented by a normal distribution, the T- Student to obtain the confidence interval of the population mean, considering a confidence level of 95%.

Considering that the best results obtained the *A. fraterculus* wing beat signal as having a fundamental frequency by the CAEA algorithm (autocorrelation method) with the population mean of 113.75 ± 2.04 Hz with a confidence level of 95%, with a dispersion given by the standard deviation of 7.97 Hz, with a slight flattening (kurtosis coefficient of −0.54) and practically symmetric (asymmetry coefficient of −0.05) with respect to a normal distribution and with values in the range of 95.52 Hz to 129.38 Hz. For measurement by fundamental frequency obtained by the location of the peak in the CAEA algorithm (FFT method), the signal has a population mean of 116.40 ± 3.10 Hz with a confidence level of 95%, with a dispersion given by the standard deviation of 10.09 Hz, with a slight flattening (kurtosis coefficient of −0.72) and slightly asymmetric (asymmetry coefficient of −0.35) with respect to a normal distribution and with values in the range of 94.00 Hz to 132.00 Hz. In the case of measurement of the frequency by the difference between the 2nd component and the fundamental frequency obtained in the CAEA algorithm (FFT method), the signal has a population mean of 110.50 ± 3.33 Hz with a confidence level of 95%, with a dispersion given by the standard deviation of 12.56 Hz, with a slight flattening (kurtosis coefficient of −0.64) and slightly asymmetric (coefficient of asymmetry of 0.19) with respect to a normal distribution and with values in the range of 84.00 Hz to 136.00 Hz.

Due to the difficulty of locating the higher frequency components, it was not possible to use their data for the characterization of the signal, since they present great variation in their minimum and maximum values. Thus, only the relationship between the magnitude of the fundamental frequency and the magnitude of 2nd component, obtaining a sample mean of 2.26, with a dispersion given by the standard deviation of 0.75, with a slight flattening (kurtosis coefficient of −0.66) and slightly asymmetric (coefficient of asymmetry of 0.44) with respect to a normal distribution and with values in the range of 1.07 to 3.92.

### 3.2. Measurement of the Wing Beat Signal Generated by *C. capitata*

**Figure 23:**
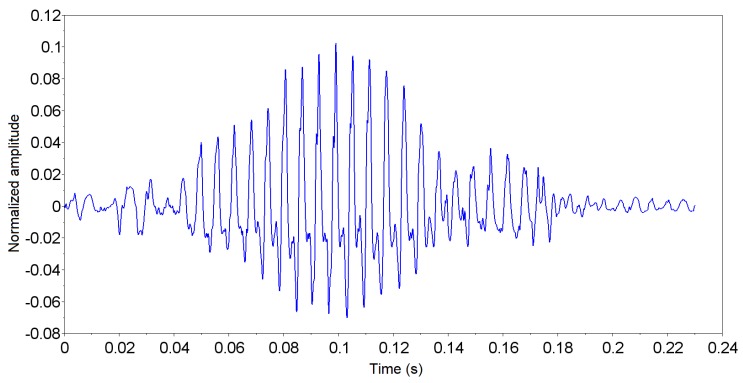
*Ceratitis capitata* passing event signal of the standard group.

In the experiments performed with *C. capitata* were recorded at about seventeen hours of signal, separated into seventeen signal tracks of one hour each, to facilitate signal processing realized. Each signal track was submitted to the PEDA algorithm, with 1010 possible events of passage. The possible localized passage events were analyzed and classified in the standard group for the characterization of the wing beat signal generated by *C. capitata*. In addition, 111 passing events were selected, (Figure 23), from the 1010 possible events of passage located (according to the criteria presented in Section 2.2).

The data obtained by extracting the characteristics of the signals from the 111 events of passage of the standard group of *C. capitata* using the CAEA algorithm were analyzed and the descriptive measures complete statistical are presented in Appendix A. Table A5 and Table A6 present the descriptive measures of the data of the complete samples. In the Table A7 and Table A8, the descriptive measures with the removal of outliers in each characteristic of the analyzed signal, we considered data outliers that are outside the minimum and maximum limits of the boxplot.

**Figure 24:**
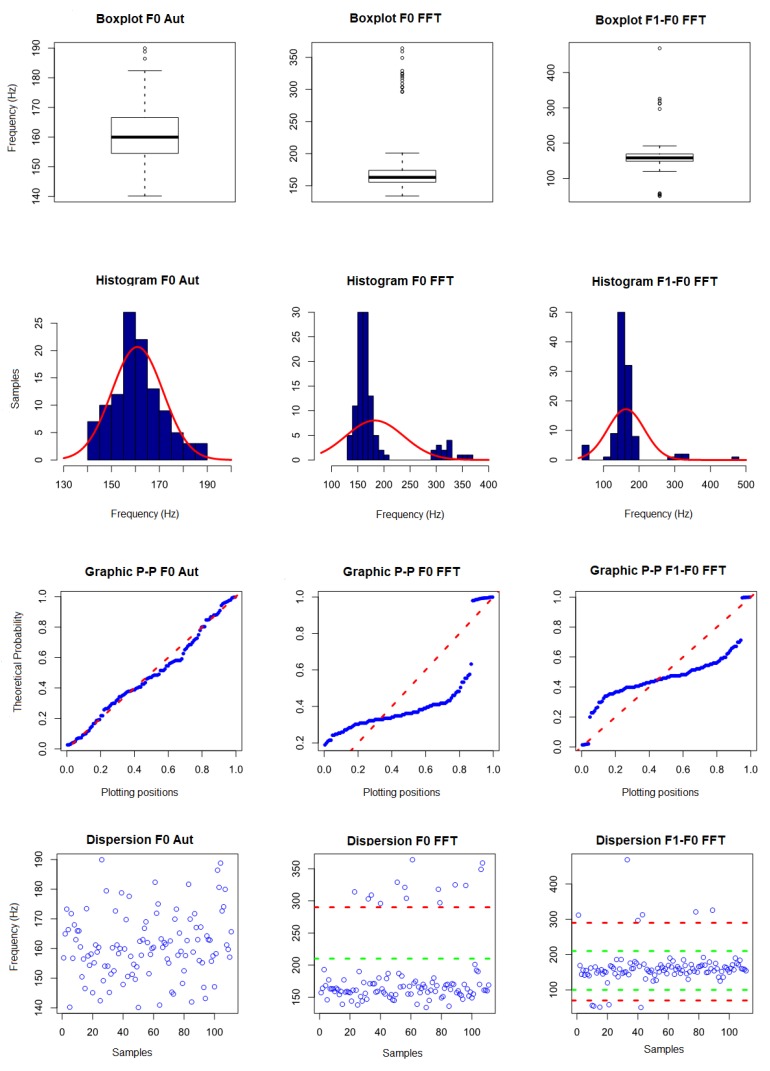
Graphics of the visual evaluation of the data obtained with the events of passage of the *C. capitata* of the standard group without removal of outliers.

In the analysis of the complete data without removal of outliers (Table A5), it was observed that, due to the degradation in the higher frequency frequencies obtained from the signals, there was a difficulty in locating the peaks corresponding to the their components. Thus, it was not possible to use the data for characterization of the signal, since they present great variation between the minimum and maximum values. Thus, the fundamental frequency obtained by the CAEA algorithm (autocorrelation method) (F0 Aut), the fundamental frequency obtained by the CAEA algorithm (FFT method) (F0 FFT) and the frequency obtained by the difference between the frequency of the 2nd component and the fundamental frequency, both obtained by the CAEA algorithm (FFT method) (F1-F0 Aut), were analyzed. Figure 24 presents the visual evaluation performed.

In the measurement of the fundamental frequency by the CAEA algorithm (autocorrelation method), three data (186.41 Hz, 188.79 Hz and 189.41 Hz) were observed above the maximum limit for outliers (184.7 Hz), in the measurement of the fundamental frequency obtained in the CAEA algorithm (FFT method), the second column, we observed 14 data (with values from 303 Hz to 364 Hz) with possibilities of being outliers (maximum limit 201.75 Hz), in the measurement of the frequency by the difference between the 2nd component and the fundamental frequency obtained in the CAEA algorithm (FFT method) were observed four data (with values from 50 Hz to 58 Hz) and six data (with values from 297 Hz to 469 Hz) with the possibility of being outliers (lower limit 117.75 Hz and upper limit 199.75 Hz). Based on the boxplot graphics, the data with removal of the outliers indicate that the possibility of being represented by a normal distribution.

In the second line of Figure 24, the histograms (blue) and curve of the superimposed normal distribution (red) are shown. With respect to the data of the first column, the highest concentration of values near the mean is observed, distribution being practically symmetrical, with the bell format, without data gaps and without outliers data, indicating a normal distribution. In the second column, we observe the displaced mean being to the right of most of the data, presenting a gap between the data with a new concentration of data (possible outliers) to the left of the mean, indicating a non-normal distribution. In the third column, we observe a higher concentration of values near the mean, having outliers data above 250 Hz and below 80 Hz, indicating a non-normal distribution.

In the third line of Figure 24, we present the P–P graphics with the probability distribution of the sample data (blue) superimposed on the probability distribution of a normal curve (red). In the first column, it is observed that the probability distribution of the data tends to follow the probability distribution of a normal curve, indicating a normal distribution. In the second column, it is observed that the probability distribution of the data does not follow the distribution of probability of a normal curve, with a gap between the data, indicating a non-normal distribution. In the third column, it is observed that the probability distribution of the data does not follow the probability distribution of a normal curve, having gaps in the lower and upper part, indicating a non-normal distribution.

As a complementary evaluation, the Shapiro–Wilk normality test was performed. A *p*-value of 0.08254 (above the 0.05 limit for normality) was obtained for fundamental frequency by the CAEA algorithm (autocorrelation method), a *p*-value of $1.33\times 10^{-15}$ (below the limit of 0.05 for normality) was obtained for fundamental frequency obtained in the CAEA algorithm (FFT method) and frequency for the difference between the 2nd component and the fundamental frequency obtained in frequency spectrum—FFT had a *p*-value of $8.442\times 10^{-15}$ (below the limit of 0.05 for normality). Based on the visual evaluation and the Shapiro–Wilk normality test, it was found that the fundamental frequency data obtained by the CAEA algorithm (autocorrelation method) can be represented by a normal distribution, the fundamental frequency data obtained by the peak location in frequency spectrum—FFT can not be represented by a normal distribution and the frequency data obtained by the difference between the frequency of the 2nd harmonic and the fundamental frequency both obtained by the location of its peaks in the CAEA algorithm (FFT method) can not be represented by the normal distribution.

With the results obtained with the normality evaluation, the visual analysis of the data dispersion (fourth line—Figure 24) was performed, it being observed in the third column that the data are grouped between 100 Hz and 200 Hz (green lines), with 100 occurrences and below 70 Hz, five occurrences, and above 280 Hz (red line), six occurrences, the data being considered outliers and can be removed from the dataset. In the second column, the data are grouped below 220 Hz (green line), with 97 occurrences and above 280 Hz (red line), with 14 occurrences. Based on the performed analyses, it is noted that data above 280 Hz represent an error in the location of the peak corresponding to the fundamental frequency, being considered outliers and retired from the set of data. With respect to the scatter plot of the first column, it was observed that the three data with the possibility of being considered outliers (186.41 Hz, 188.79 Hz and 189.41 Hz) are close to the maximum limit obtained by boxpot (184.7 Hz) and do not present discrepancies with the the dispersion pattern presented by the remainder of the data, carrying were not considered outliers. The fundamental frequency obtained by the CAEA algorithm (autocorrelation method) presented evaluation errors, in which case the measured value is below the expected value.

**Figure 25:**
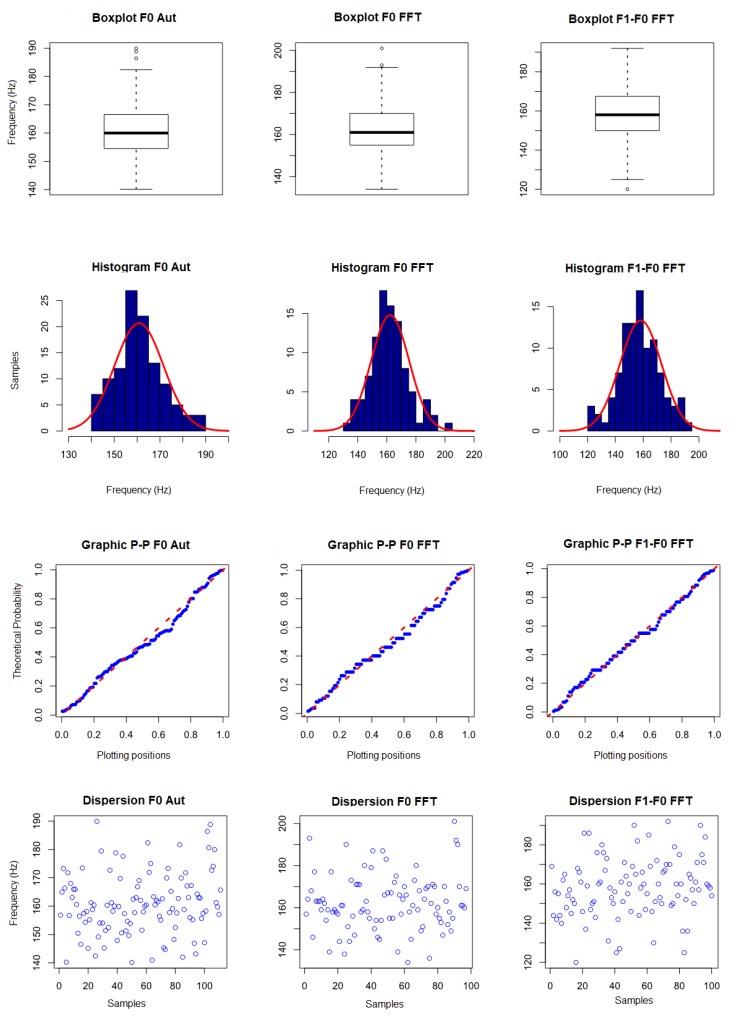
Graphics of the visual evaluation of the data obtained with the events of passage of *C. capitata* of the standard group without outliers.

Figure 25 presents the visual evaluation of data with outliers removal. Each column, from left to right, presents the data for fundamental frequency measurements obtained by the CAEA algorithm (autocorrelation method), fundamental frequency obtained by the location of the peak in the CAEA algorithm (FFT method) and the frequency obtained by the difference between the frequency of the 2nd component and the fundamental frequency both obtained by the location of their peaks in the CAEA algorithm (FFT method). The results for the fundamental frequency obtained by the CAEA algorithm (autocorrelation method) were not changed. Once they did not have outliers data, they were kept for comparison with the data obtained with the removal of the outliers of the fundamental frequency obtained in the CAEA algorithm (FFT method) and the frequency by the difference between the 2nd component and the fundamental frequency obtained in the CAEA algorithm (FFT method).

In the first line of Figure 25, the boxplot graphics are shown. In the data concerning the fundamental frequency measurement obtained in the CAEA algorithm (FFT method), the second column, a slightly asymmetrical distribution is observed with the median line near the lower part of the box, slightly asymmetrical mustaches and slightly longer than the subsections of the box and with two data (201 Hz and 193 Hz) slightly above the upper limit (192 Hz) not being considered outliers, indicating that this distribution may be normal. In the data concerning the fundamental frequency of the difference between the 2nd component and the fundamental frequency obtained in the CAEA algorithm (FFT method), the third column, a symmetrical distribution is observed, symmetrical mustaches and slightly longer than the subsections of the box and with a die (120 Hz) slightly below the lower limit (124 Hz) not being considered outliers, indicating that this distribution may be normal. In relation to the fundamental frequency boxplot by the CAEA algorithm (autocorrelation method), the first column, the graphics present similarities in their dispersions, with averages and medians nearby.

In the second line of Figure 25, the histograms (blue) and curve of the superimposed normal distribution (red) are shown. In the second column, we observe the highest concentration of values near the mean with a longer tail on the right, with bell shape, with a small gap above 200 Hz and without outliers data, indicating a normal distribution. In the third column, we observe the highest concentration of values close to the mean with symmetrical distribution, with the bell format, with no data gaps and no outliers data, indicating a normal distribution. In comparison with the histogram of the first column, the similarity between its distributions is observed, being that in the second and third columns the distribution is wider.

In the third line of Figure 22, we present the P–P graphics with the probability distribution of the sample data (blue) superimposed on the probability distribution of a normal curve (red). In the second and third columns, it is observed that the probability distribution of the data tends to follow the probability distribution of a normal curve, indicating that both can be represented by a normal distribution.

The Shapiro–Wilk normality test was realized as a normality complementary evaluation being obtained a *p*-value of 0.09642 (above the 0.05 limit for normality) for the fundamental frequency by the CAEA algorithm (FFT method) and a *p*-value of 0.5932 (above the limit of 0.05 for normality) for the frequency obtained by the difference between the 2nd component and the fundamental frequency by the CAEA algorithm (FFT method). Based on the visual evaluation and the Shapiro–Wilk normality test, it was observed that the fundamental frequency data obtained by the peak location in the CAEA algorithm (FFT method), considering the erroneous detections as outliers, can be represented by a normal distribution and the frequency data obtained by the difference between the frequency of the 2nd component and the fundamental frequency both obtained by the location of its peaks in the CAEA algorithm (FFT method), without the outliers data, can be represented by the normal distribution.

Due to sample sizes (111 for fundamental frequency by the CAEA algorithm (autocorrelation method), 100 for fundamental frequency by the CAEA algorithm (FFT method) and 97 for frequency by difference between peaks by the CAEA algorithm (FFT method)) and that the data can be represented by a normal distribution, the T-Student to obtain the confidence interval of the population mean, considering a confidence level of 95%.

Considering the best results obtained the wing beat signal generated by *C. capitata* have a fundamental frequency by the CAEA algorithm (autocorrelation method) with the population mean of 160.81±2.02 Hz with a confidence level of 95%, with a dispersion given by the standard deviation of 10.71 Hz, slightly accentuated (kurtosis coefficient of 0.11) and slightly asymmetrical (asymmetry coefficient of 0.41) with respect to a normal distribution and with values in the range of 140.15 Hz to 189.91 Hz. For measurement by the fundamental frequency obtained by location of the peak using the CAEA algorithm (FFT method), the signal has a population mean of 162.25 ± 2.63 Hz with a confidence level of 95%, with a dispersion given by the standard deviation of 13.06 Hz, slightly accentuated (kurtosis coefficient of 0.33) and slightly asymmetric coefficient (asymmetry coefficient of 0.44) with respect to a normal distribution and with values in the range of 134.00 Hz to 201.00 Hz. The frequency by the difference between the 2nd component and the fundamental frequency obtained in the CAEA algorithm (FFT method) it has a population mean of 158.00 ± 2.97 Hz with a confidence level of 95%, with a dispersion given by the standard deviation of 14.95 Hz, with a slight flattening (kurtosis coefficient of -0.03) and slightly asymmetrical (asymmetry coefficient of −0.04) with respect to a normal distribution and with values in the range of 120.00 Hz to 192.00 Hz.

Due to the difficulty of locating the higher frequency components, it was not possible to use their data for the characterization of the signal, since they present great variation in their minimum and maximum values. Thus, it was analyzed only the relationship between the magnitude of the fundamental frequency and the magnitude of the frequency by the difference between the 2nd component and the fundamental frequency, both obtained by the CAEA algorithm (FFT method), being obtained a sample mean of 2.05, with a dispersion given by the standard deviation of 0.96, with an accentuation (kurtosis coefficient of of 2.93) and slightly asymmetric (coefficient of asymmetry of 1.61) in relation to a normal distribution and with values in the range of 1.01 to 6.06.

### 3.3. Analysis of the Wing Beat Signal Generated by *A. fraterculus* and *C. capitata*

In the analysis of the wing beat signal generated by *A. fraterculus* and *C. capitata* were utilized the signal characteristics obtained through the CAEA algorithms-autocorrelation method (fundamental frequency), CAEA-FFT method (fundamental frequency) and CAEA-FFT method (frequency measured by the difference between the fundamental frequency and the frequency of the 2nd harmonic).

**Figure 26:**
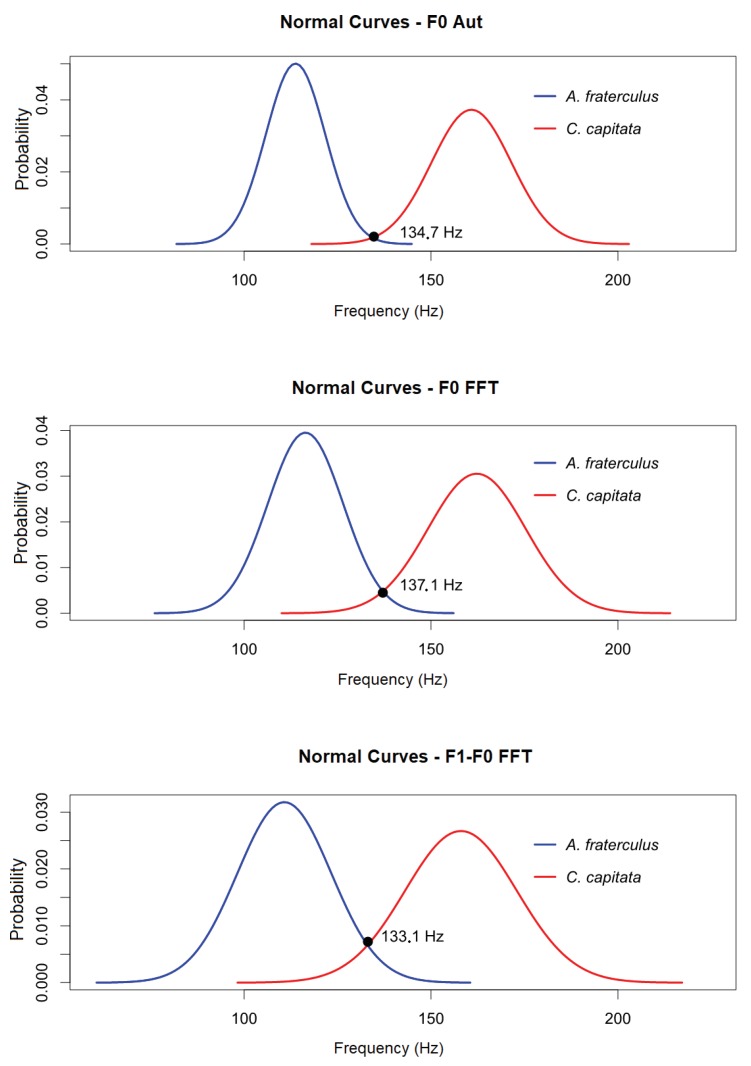
Comparison between the normal curves for fundamental frequency by the CAEA algorithm (autocorrelation method) (F0 Aut.), fundamental frequency by the CAEA algorithm (FFT method) (F0 FFT) and frequency by the difference between the frequencies of the 2nd component and the fundamental frequency by the CAEA algorithm (FFT method) (F1-F0 FFT) of the events of passage of *C. capitata* of the standard group and *A. fraterculus* of the standard group.

Figure 26 presents the comparison between the normal curves for the fundamental frequencies for *A. fraterculus* and *C. capitata* obtained through the CAEA algorithm (autocorrelation method), location of the peak in the CAEA algorithm (FFT method) and the frequency relation between the peak of the 2nd component and the peak of the fundamental frequency in the CAEA algorithm (FFT method). Note that a distinction is made between the two species in relation to the fundamental frequency of wing beat and the difference frequency between the 2nd component and the fundamental frequency, despite the overlap in the limit of the normal distributions.

From the evaluation performed, it is possible to obtain the probability that a passing event of *A. fraterculus* is identified as *C. capitata*, or vice versa, calculating the cumulative probability for a normal distribution based on the intersection of the curves. With this overlap, a probability of 0.0042 was obtained for the fundamental frequency by the CAEA algorithm (autocorrelation method) that an event of *A. fraterculus* is identified as *C. capitata* and a probability of 0.0073 that a *C. capitata* event is identified as *A. fraterculus*. For the evaluation with the fundamental frequency obtained by location of the peak in the CAEA algorithm (FFT method), it was obtained a probability of 0.0201 that an event of *A. fraterculus* is identified as *C. capitata* and a probability of 0.0270 that a *C. capitata* event is identified as *A. fraterculus*. In the case of the evaluation with the frequency obtained by the relation between the peak of the 2nd component and the fundamental frequency peak in the CAEA algorithm (FFT method), a probability of 0.0375 was obtained that an event of *A. fraterculus* is identified as *C. capitata* and a probability of 0.0479 that an event of *C. capitata* be identified as *A. fraterculus*.

Regarding the analysis of data concerning the magnitudes, *C. capitata* has a mean magnitude ratio of 1.79 with a dispersion given by the standard deviation of 0.64 and *A. fraterculus* has a mean magnitude ratio of 2.05 with one dispersion given by the standard deviation of 0.65. Due to the overlapping of values, it is not possible to use the relationship between the magnitude of the fundamental frequency and the magnitude of the 2nd component for species recognition.

Analyzing the signs of events of passage with the methods of the CAEA algorithm, it was possible to extract the characteristics concerning the fundamental frequency (autocorrelation and FFT methods), frequency of the 2nd component (FFT method), the magnitude of the fundamental frequency (FFT method) and magnitude of the 2nd component (FFT method). It was observed that the use of phototransistors as receiving elements did not allow the correct evaluation of the characteristics referring to the 3rd to 5th components. This occurred due to the degradation of the signal spectrum in the upper frequencies that made it difficult to correctly locate the peaks corresponding to the components. This problem was also observed in [11].

With the characteristics of the extracted signal, the fundamental frequency of the wing beat of fruit flies was obtained using the value obtained by the CAEA algorithm (autocorrelation and FFT methods) and the difference between the frequencies of the 2nd component and the fundamental frequency by the CAEA algorithm (FFT method). Observing the statistical measures performed and the probability of identification errors occurring among the fruit flies analyzed, it was verified that the most effective method to obtain the fundamental frequency of the signal generated by the wing beat is CAEA algorithm (autocorrelation method) followed by obtaining the CAEA algorithm (FFT method) and finally that the frequency measurement of the difference between the 2nd component and the fundamental frequency presented the worst result for the classification.

However, it was observed that all three methods have measurement errors. The CAEA algorithm (autocorrelation method) presented erroneous measurements with values corresponding to half of the expected value for the fundamental frequency.The CAEA algorithm (FFT method) presented erroneous measures for the fundamental frequency with values corresponding to twice the correct fundamental frequency (next to what would be the frequency of the second component). For the frequency measurement of the difference between the 2nd component and the fundamental frequency, the CAEA algorithm (FFT method) presented values below and above that expected for the fundamental frequency. With these measurement errors, an *A. fraterculus* can be incorrectly identified as a *C. capitata* using the fundamental frequency obtained by the CAEA algorithm (FFT method), once this error indicates a frequency close to the fundamental frequency of *C. capitata*, as well as the measurement of the fundamental frequency by the CAEA algorithm (autocorrelation method) of a *C. capitata*, may present an error that approximately corresponds to the fundamental frequency of *A. fraterculus*. Therefore, to minimize fundamental frequency measurement errors, the best results are obtained by using the three methods together.

### 3.4. Dataset

The dataset obtained by the wing beat signal generated by *A. fraterculus* contains 17 h of raw signal, separated in signal tracks of one hour each, with easier signal processing software. With the analysis through the PEDA algorithm, 466 events of passage were located. Being that 66 of these events were labeled for the standard group and analyzed in the characterization of the signal corresponding to the wing beat of *A. fraterculus* using the CAEA algorithm, the remaining 400 events were not analyzed in the scope this work. For the 66 events of passage analyzed, it was possible to measure the fundamental frequency of the signal by the CAEA algorithm (autocorrelation method) in all. For the fundamental frequency obtained by location of the peak in the CAEA algorithm (FFT method), it was possible to perform the measurement in 48 events and 18 events presented measurement errors with values above 200 Hz. In the case of the frequency obtained by the difference between the frequency of the 2nd component and the fundamental frequency both obtained by the location of its peaks in the CAEA algorithm (FFT method), it was possible to perform the measurement in 62 events, and four events presented measurement errors with values above 200 Hz. Values with measurement errors were considered outliers and taken from the dataset for characterization of the wing beat signal generated by *A. fraterculus*.

The dataset obtained by the wing beat signal generated by *C. capitata* contains 17 h of raw signal, separated in signal tracks of one hour each, with easier signal processing software. With the analysis through the PEDA algorithm, 1010 events of passage were located. Being that 111 of these events were labeled for the standard group and analyzed in the characterization of the signal corresponding to the wing beat of *C. capitata*, the remaining 899 events were not analyzed in the scope of this work. For the 111 events of passage analyzed, it was possible to measure the fundamental frequency of the signal by the CAEA algorithm (autocorrelation method) in all. For the fundamental frequency obtained by location of the peak in the CAEA algorithm (FFT method), it was possible to perform the measurement in 97 events and 14 events presented measurement errors with values above 280 Hz. In the case of the frequency obtained by the difference between the frequency of the 2nd component and the fundamental frequency both obtained by the location of its peaks in the CAEA algorithm (FFT method), it was possible to perform the measurement in 100 events, six events presented measurement errors with values above 280 Hz and five events presented measurement errors with values below 70 Hz. Values with measurement errors were considered outliers and taken from the dataset for characterization of the wing beat signal generated by *C. capitata*.

## 4. Conclusions

This work presented a study and development of a real-time optoelectronic detection of insects. Based on the study, an optoelectronic sensor was developed to be used in the detection of fruit fly species *A. fraterculus* and *C. capitata*.

Regarding the fruit fly experiments, the characterization of the wing beat signal generated by *A. fraterculus* and *C. capitata* was performed using the developed optoelectronic sensor. For *A. fraterculus*, the fundamental frequency of the wing beat signal was determined with 113.75 ± 2.04 Hz with a confidence level of 95%, with a dispersion given by the standard deviation of 7.97 Hz. *C. capitata* presenting a fundamental frequency of the wing beat signal generated with the value of 160.81 ± 2.02 Hz with a confidence level of 95%, with a dispersion given by the standard deviation of 10.71 Hz. Both results were obtained with the CAEA algorithm (autocorrelation method), which was considered the most effective method for the extraction of characteristics.

A dataset of the wing beat signal generated by *A. fraterculus* and *C. capitata* captured by the developed optoelectronic sensor was elaborated. The *A. fraterculus* dataset has 17 h of raw signal recording, separated in one-hour signal tracks with 466 event-of-passage signals located through the PEDA algorithm (RMS method). Of these, 66 events were selected and analyzed for signal characterization generated by the beat wings of *A. fraterculus*. For *C. capitata*, the dataset has 17 h of raw signal recording separated in one-hour signal tracks with 1010 event-passing signals located through the PEDA algorithm (RMS method). Of these, 111 events were selected and analyzed for the wing beat signal generated by *C. capitata*.

Finally, with the strong evidence that the optoelectronic sensor presented can be used in an intelligent trap, the authors expect to build, deploy and collect field data to extend the findings of this work. Future work includes improvements in the sensor for lower power, analyzing the wing beat signal generated by wild flies, better detection characteristics, evaluating classifiers for be applied to insect recognition, telemetry and in the years following, collecting data currently not available in part of the globe.

## Abbreviations

The following abbreviations are used in this manuscript:

RMS	Root mean square
Aut	Autocorrelation
DC	Direct current
FFT	Fast Fourier transform
F0	Fundamental frequency
F1	2nd component frequency
F2	3rd component frequency
F3	4th component frequency
F4	5th component frequency
M0	Fundamental frequency magnitude
M1	2nd component magnitude
M2	3rd component magnitude
M3	4th component magnitude
M4	5th harmonic magnitude
$\bar{X}$	Arithmetic mean
$S_{x}$	Sample standard error
*S*	Sample standard deviation
S(%)	Sample standard deviation percentage
*K*	Kurtosis coefficient
As	Coefficient of asymmetry
*H*	Sample range
Min	Minimum sample value
Max	Maximum sample value
LED-IR	Infrared light emitting diode
MFB	Multiple feedback
Mag	Magnitude
CAEA	Algorithm of automatic extraction of characteristics
PEDA	Algorithm of detection of events of passage

## Appendix A. Statistical Descriptive Measures of the Data Concerning the Standard Group of *A. fraterculus* and *C. capitata*

The statistical descriptive measures of the frequencies and magnitude characteristics of the wing beat signal generated by *A. fraterculus* are presented in Table A1 and Table A2 (complete data) and in Table A3 and Table A4 (outlier values removed). Table A5 and Table A6 (complete data) and Table A7 and Table A8 (outliers values removed) present the statistical descriptive measures of the frequency and magnitude characteristics of the wing beat signal generated by *C. capitata*.

**Table A1:** Statistical descriptive measures of the frequency data of the standard group of *A. fraterculus*.

	$\bar{X}\left(\mathrm{Hz}\right)$	$S_{x}\left(\mathrm{Hz}\right)$	S(Hz)	S(%)	*K*	As	H(Hz)	Min(Hz)	Max(Hz)
F0 Aut.	113.75	1.02	7.97	7.01	−0.54	−0.05	33.86	95.52	129.38
F0 FFT	146.46	6.14	47.93	32.72	−1.06	0.86	148.00	94.00	242.00
F1 FFT	264.49	7.99	62.38	23.59	2.04	1.46	284.00	192.00	476.00
F2 FFT	391.59	10.71	83.63	21.36	3.99	1.53	462.00	272.00	734.00
F3 FFT	512.90	13.22	103.23	20.13	0.92	0.92	499.00	332.00	831.00
F4 FFT	630.13	14.41	112.52	17.86	0.65	0.63	581.00	390.00	971.00
F1-F0 FFT	118.03	4.00	31.28	26.50	7.78	2.76	155.00	84.00	239.00
F2-F0 FFT	245.13	8.26	64.53	26.33	3.98	1.88	352.00	153.00	505.00
F3-F0 FFT	366.44	10.39	81.13	22.14	0.23	0.81	389.00	213.00	602.00
F4-F0 FFT	483.67	11.61	90.70	18.75	0.23	0.38	471.00	271.00	742.00

**Table A2:** Statistical descriptive measures of the magnitude data of the standard group of *A. fraterculus*.

	$\bar{X}\left(\mathrm{Mag}\right)$	$S_{x}\left(\mathrm{Mag}\right)$	S(Mag)	S(%)	*K*	As	H(Mag)	Min(Mag)	Max(Mag)
M0-FFT	536.12	28.48	222.43	41.49	3.14	1.54	1148.92	245.60	1394.52
M1-FFT	249.34	16.12	125.91	50.50	2.28	1.37	589.54	68.08	657.63
M2-FFT	116.93	5.49	42.87	36.66	2.40	0.97	242.13	31.57	273.69
M3-FFT	74.33	3.56	27.83	37.44	−0.50	0.53	120.17	18.97	139.14
M4-FFT	50.62	2.58	20.13	39.77	−0.10	0.62	87.46	10.99	98.45
M0/M1	2.41	0.12	0.92	38.29	−0.06	0.76	3.58	1.07	4.66
M0/M2	4.86	0.24	1.84	37.83	0.93	1.17	7.93	2.54	10.46
M0/M3	7.73	0.39	3.05	39.45	1.43	1.18	14.84	3.15	17.99
M0/M4	11.65	0.66	5.13	44.07	3.10	1.47	27.47	3.93	31.39
M1/M2	2.16	0.09	0.74	34.31	−0.49	0.44	2.99	1.01	4.00
M2/M3	1.64	0.07	0.51	31.09	12.07	2.58	3.33	1.01	4.34
M3/M4	1.53	0.05	0.38	24.81	0.04	0.87	1.52	1.01	2.53

**Table A3:** Statistical descriptive measures of data without outliers by frequency measurement of the standard group of *A. fraterculus*.

	$\bar{X}\left(\mathrm{Hz}\right)$	$S_{x}\left(\mathrm{Hz}\right)$	S(Hz)	S(%)	*K*	As	H(Hz)	Min(Hz)	Max(Hz)
F0 Aut.	113.75	1.02	7.97	7.01	−0.54	−0.05	33.86	95.52	129.38
F0 FFT	116.4	1.53	10.09	8.67	−0.72	−0.35	148.00	94.00	132.00
F1 FFT	258.66	7.09	54.47	21.06	2.80	1.47	284.00	192.00	476.00
F2 FFT	381.75	8.37	64.30	16.84	−0.84	0.37	264.00	272.00	536.00
F3 FFT	496.02	10.96	82.76	16.69	0.63	0.53	430.00	332.00	762.00
F4 FFT	626.63	13.51	103.78	16.56	0.06	0.35	488.00	390.00	878.00
F1-F0 FFT	110.50	1.66	12.56	11.36	-0.64	0.19	155.00	84.00	136.00
F2-F0 FFT	237.80	6.60	50.73	21.33	1.19	1.35	227.00	153.00	380.00
F3-F0 FFT	359.42	9.44	72.47	20.16	-0.30	0.60	307.00	213.00	520.00
F4-F0 FFT	477.56	10.20	77.04	16.13	-0.46	0.30	321.00	322.00	643.00

**Table A4:** Statistical descriptive measures of the data without outliers by magnitude measurement of the standard group of *A. fraterculus*.

	$\bar{X}\left(\mathrm{Mag}\right)$	$S_{x}\left(\mathrm{Mag}\right)$	S(Mag)	S(%)	*K*	As	H(Mag)	Min(Mag)	Max(Mag)
M0-FFT	536.12	28.48	222.43	41.49	3.14	1.54	1148.92	245.60	1394.52
M1-FFT	249.34	16.12	125.91	50.50	2.28	1.37	589.54	68.08	657.63
M2-FFT	116.93	5.49	42.87	36.66	2.40	0.97	242.13	31.57	273.69
M3-FFT	74.33	3.56	27.83	37.44	−0.50	0.53	120.17	18.97	139.14
M4-FFT	50.62	2.58	20.13	39.77	−0.10	0.62	87.46	10.99	98.45
M0/M1	2.26	0.10	0.75	33.26	−0.66	0.44	2.85	1.07	3.92
M0/M2	4.54	0.19	1.41	31.14	0.22	0.83	5.67	2.54	8.20
M0/M3	7.42	0.33	2.56	34.53	−0.19	0.72	10.30	3.15	13.45
M0/M4	11.04	0.59	4.48	40.58	6.29	1.75	27.47	3.93	31.39
M1/M2	2.10	0.09	0.68	32.26	−0.87	0.26	2.58	1.01	3.59
M2/M3	1.60	0.05	0.37	23.38	−0.05	0.45	1.53	1.01	2.54
M3/M4	1.51	0.05	0.36	23.74	−0.11	0.81	1.39	1.01	2.39

**Table A5:** Statistical descriptive measures of the frequency data of the standard group of *C. capitata*.

	$\bar{X}\left(\mathrm{Hz}\right)$	$S_{x}\left(\mathrm{Hz}\right)$	S(Hz)	S(%)	*K*	As	H(Hz)	Min(Hz)	Max(Hz)
F0 Aut.	160.81	1.02	10.71	6.66	0.11	0.41	49.76	140.15	189.91
F0 FFT	182.43	5.24	55.26	30.29	3.29	2.15	230.00	134.00	364.00
F1 FFT	345.68	7.98	84.07	24.32	3.82	1.80	461.00	190.00	651.00
F2 FFT	520.50	10.76	113.31	21.77	1.94	0.95	659.00	300.00	959.00
F3 FFT	690.60	13.81	145.51	21.07	0.44	0.51	748.00	362.00	1110.00
F4 FFT	834.58	15.81	166.62	19.96	−0.07	0.31	759.00	484.00	1243.00
F1-F0 FFT	163.25	4.89	51.52	31.56	13.42	2.63	419.00	50.00	469.00
F2-F0 FFT	338.06	8.49	89.40	26.44	1.43	0.79	487.00	154.00	641.00
F3-F0 FFT	508.17	11.64	122.61	24.13	0.68	0.40	661.00	223.00	884.00
F4-F0 FFT	652.14	13.95	146.93	22.53	0.23	0.31	716.00	326.00	1042.00

**Table A6:** Statistical descriptive measures of the magnitude data of the standard group of *C. capitata*.

	$\bar{X}\left(\mathrm{Mag}\right)$	$S_{x}\left(\mathrm{Mag}\right)$	S(Mag)	S(%)	*K*	As	H(Mag)	Min(Mag)	Max(Mag)
M0-FFT	428.58	14.03	147.86	34.50	0.41	0.61	767.45	157.28	924.73
M1-FFT	237.45	11.51	121.26	51.07	2.77	1.42	678.26	70.60	748.86
M2-FFT	103.54	4.69	49.41	47.72	2.35	1.40	267.13	22.20	289.33
M3-FFT	64.98	3.29	34.64	53.31	1.89	1.32	181.68	17.23	198.91
M4-FFT	42.16	2.00	21.07	49.97	1.16	1.14	105.40	13.11	118.51
M0/M1	2.10	0.10	1.03	48.80	2.25	1.54	5.06	1.01	6.06
M0/M2	4.74	0.21	2.21	46.70	3.75	1.60	13.06	1.43	14.49
M0/M3	7.86	0.37	3.89	49.47	2.60	1.47	20.77	2.39	23.16
M0/M4	11.85	0.53	5.55	46.79	2.85	1.42	32.14	2.81	34.96
M1/M2	2.50	0.11	1.20	47.98	3.13	1.48	6.78	1.00	7.78
M2/M3	1.74	0.06	0.66	37.95	2.84	1.59	3.46	1.00	4.46
M3/M4	1.59	0.05	0.51	32.25	2.83	1.46	2.71	1.02	3.73

**Table A7:** Statistical descriptive measures of data without outliers by frequency measurement of the standard group of *C. capitata*.

	$\bar{X}\left(\mathrm{Hz}\right)$	$S_{x}\left(\mathrm{Hz}\right)$	S(Hz)	S(%)	*K*	As	H(Hz)	Min(Hz)	Max(Hz)
F0 Aut.	160.81	1.02	10.71	6.66	0.11	0.41	49.76	140.15	189.91
F0 FFT	162.25	1.33	13.06	8.05	0.33	0.44	67.00	134.00	201.00
F1 FFT	329.14	5.37	54.79	16.65	2.34	0.91	295.00	190.00	485.00
F2 FFT	510.39	9.26	96.24	18.86	−0.36	0.22	441.00	300.00	741.00
F3 FFT	668.55	11.84	120.79	18.07	−0.01	0.00	589.00	362.00	951.00
F4 FFT	820.23	14.67	151.79	18.51	−0.20	0.09	702.00	484.00	1186.00
F1-F0 FFT	158.00	1.49	14.95	09.45	−0.03	−0.04	72.00	120.00	192.00
F2-F0 FFT	331.74	7.84	81.50	24.57	1.31	0.58	473.00	154.00	627.00
F3-F0 FFT	494.83	10.03	102.32	20.68	0.50	0.03	549.00	223.00	772.00
F4-F0 FFT	645.22	12.40	127.10	19.70	0.02	0.19	603.00	353.00	956.00

**Table A8:** Statistical descriptive measures of the data without outliers by magnitude measurement of the standard group of *C. capitata*.

	$\bar{X}\left(\mathrm{Mag}\right)$	$S_{x}\left(\mathrm{Mag}\right)$	S(Mag)	S(%)	*K*	As	H(Mag)	Min(Mag)	Max(Mag)
M0-FFT	428.58	14.03	147.86	34.50	0.41	0.61	767.45	157.28	924.73
M1-FFT	237.45	11.51	121.26	51.07	2.77	1.42	678.26	70.60	748.86
M2-FFT	103.54	4.69	49.41	47.72	2.35	1.40	267.13	22.20	289.33
M3-FFT	64.98	3.29	34.64	53.31	1.89	1.32	181.68	17.23	198.91
M4-FFT	42.16	2.00	21.07	49.97	1.16	1.14	105.40	13.11	118.51
M0/M1	2.05	0.09	0.96	46.99	2.93	1.61	5.06	1.01	6.06
M0/M2	4.43	0.16	1.66	37.57	0.19	0.69	7.62	1.43	9.05
M0/M3	7.32	0.29	3.02	41.19	0.70	0.89	15.22	2.39	17.61
M0/M4	11.36	0.45	4.71	41.45	1.21	0.97	24.12	2.81	26.94
M1/M2	2.31	0.09	0.90	38.98	0.23	0.72	3.86	1.00	4.87
M2/M3	1.67	0.06	0.58	34.50	4.73	1.74	3.46	1.00	4.46
M3/M4	1.56	0.04	0.46	29.54	1.51	1.16	2.34	1.02	3.36
